# Terpene produced by coexpression of the TPS and P450 genes from *Lavandula angustifolia* protects plants from herbivore attacks during budding stages

**DOI:** 10.1186/s12870-023-04490-7

**Published:** 2023-10-09

**Authors:** Zhengyi Ling, Jingrui Li, Yanmei Dong, Wenying Zhang, Hongtong Bai, Shu Li, Su Wang, Hui Li, Lei Shi

**Affiliations:** 1 State Key Laboratory of Plant Diversity and Specialty Crops, Institute of Botany, Chinese Academy of Sciences, Beijing, 100093 China; 2 China National Botanical Garden, Beijing, 100093 China; 3 University of Chinese Academy of Sciences, Beijing, 100049 China; 4 Institute of Plant and Environment Protection, Beijing Academy of Agricultural and Forestry Science, Beijing, 100097 China

## Abstract

To deter herbivore attacks, plants employ a diverse array of volatile compounds, particularly during the early developmental stages. The highly expressed genes *LaTPS7*, *LaTPS8*, and *LaCYP71D582* were identified during the budding phases of *Lavandula angustifolia*. In vitro studies revealed that LaTPS7 generated nine distinct compounds, including camphene, myrcene, and limonene. LaTPS8 enzymatically converted eight volatiles by utilizing geranyl diphosphate and nerolidyl diphosphate as substrates. Overexpression of plastid-localized LaTPS7 in *Nicotiana benthamiana* resulted in the production of limonene. Furthermore, the endoplasmic reticulum-associated enzyme LaCYP71D582 potentially converted limonene into carveol. In *N. benthamiana*, LaTPS8 is responsible for the synthesis of α-pinene and sylvestrene. Furthermore, leaves transfected with LaTPS7 and leaves cotransfected with LaTPS7 and LaCYP71D582 exhibited a repellent effect on aphids, with an approximate rate of 70%. In comparison, leaves with an empty vector displayed a repellent rate of approximately 20%. Conversely, tobacco leaves expressing LaTPS7 attracted ladybugs at a rate of 48.33%, while leaves coexpressing LaTPS7 and LaCYP71D582 attracted ladybugs at a slightly higher rate of 58.33%. Subsequent authentic standard tests confirmed that limonene and carveol repel *Myzus persicae* while attracting *Harmonia axyridis*. The promoter activity of *LaTPS7* and *LaCYP71D582* was evaluated in *Arabidopsis thaliana* using GUS staining, and it was observed that wounding stimulated the expression of *LaTPS7*. The volatile compounds produced by LaTPS7, LaTPS8, and LaCYP71D582 play a crucial role in plant defence mechanisms. In practical applications, employing biological control measures based on plant-based approaches can promote human and environmental health.

**Supplementary Information:**

The online version contains supplementary material available at 10.1186/s12870-023-04490-7.

## Introduction

Plants engage in multifaceted interactions with their environment through the emission of a diverse array of volatiles, which serve various physiological and ecological functions. The hypothesis of herbivore-induced plant volatiles (HIPVs) was proposed by Ehrlich and Raven [1], suggesting that plants utilize volatiles for communication and defence against herbivore attacks. A recent investigation conducted by Liu [2] discovered a negative correlation between the production of volatile compounds in cotton plants and the presence of *Helicoverpa armigera* eggs, indicating the crucial role of volatiles in pest protection. Numerous plant-derived volatile chemicals, particularly terpenes/terpenoids, have demonstrated protective effects. For instance, farnesene acts as a repellent for aphids (*Myzus persicae*) while attracting predatory ladybugs (*Harmonia axyridis*) [3, 4]. Linalool, another common compound found in flowers and fruits, has been observed to attract thrips in *Arabidopsis thaliana*, while linalool oxides (catalysed by CYP71D) repel them [5]. Moreover, HIPVs, including volatiles released by mint plants such as limonene, 1,8-cineole, and carvone, serve as attractants for herbivore-preying animals [6]. Thus, volatile compounds can be considered part of a defence system employed by plants to protect themselves through tritrophic interactions, either directly or indirectly [7]. Furthermore, it has been observed that volatile chemicals are likely expressed at higher concentrations during early developmental stages or in specific tissues, with subsequent maintenance at consistent levels and reduction during mature or senescence phases [8]. This dynamic regulation of volatile chemical expression in plants enables them to adapt to their surroundings and ensure successful reproduction.

The majority of HIPVs are composed of terpenes/terpenoids, which are synthesized through the mevalonate (MVA) pathway, localized in the cytosol and producing sesquiterpenes, and the 2-C-methyl-D-erythritol-4-phosphate (MEP) pathway, occurring in plastids and producing monoterpene. Within the plant kingdom, terpene synthases (TPSs) form a medium-sized gene family comprising various subfamilies. Moreover, investigations have extensively explored the structure and function of terpenes in peppermint (*Mentha×piperita*) [9], *Artemisia annua* L. [10], and *A. thaliana*, particularly regarding the postprocessing of terpenes by coexpressed genes [5]. However, it remains unknown whether similar catalytic mechanisms and corresponding ecological functions occur in *Lavandula angustifolia*, a terpene-rich plant, and which specific genes are coexpressed to regulate the production of volatile terpenes/terpenoids in this species.

*L. angustifolia*, commonly known as lavender, is a flowering plant renowned for its production of approximately 70 volatile chemicals within its blossoms, such as limonene, *α*-pinene, and linalool, among others. Due to its intricate terpene biosynthesis regulation, lavender has emerged as a prominent model plant for studying this aspect [11]. Through in vitro experimentation, specific enzymes involved in terpene production in lavender have been identified, including LaLIMS, LaLINS, *τ*-cadinol synthase, 1,8-cineole synthases and *β*-phellandrene synthase [12–15]. Despite the well-established requirement of terpene synthases (TPSs) and cytochrome P450 (CYP) enzymes for terpene and terpene derivative biosynthesis [16] and the fact that CYP family members constitute approximately 1% of the genome in most plant species [17], the functional characterization of CYP genes in lavender remains unexplored.

The physiological activities of volatiles and the genes involved in tritrophic interactions in lavender remain largely unknown. Previous investigations have explored the composition of volatile organic compounds (VOCs) during inflorescence ontogeny, revealing the categorization of terpenes into three distinct groups across three flowering developmental phases [18]. Notably, limonene falls within the first group and exhibits natural insect repellent properties. Our recent study identified aphids and ladybugs as the most prevalent insects in lavender fields during early spring, suggesting the existence of tritrophic interactions involving volatile compounds, predators, and prey [19]. Moreover, Y-tube olfactometer trials demonstrated that *β*-trans-ocimene and (+)-*R*-limonene effectively repelled 74.71% and 78.41% of aphids, respectively [19]. Therefore, further investigation into the response of these chemicals to herbivores and the underlying gene-level mechanisms governing external stimuli is warranted to shed light on the intricate plant-insect relationship.

In this study, we cloned and identified the terpene synthase (TPS) and cytochrome P450 (CYP) genes expressed in immature lavender flowers using transcriptome data. Subsequently, we conducted in vitro and in vivo experiments to evaluate the functionality of the gene products. Additionally, we investigated the responsiveness of these genes to environmental stimuli and examined their regulatory elements. Furthermore, authentic standards were employed to assess the effects of the identified volatile compounds on aphids and ladybugs, thus elucidating their ecological potential. The outcomes of this study not only enhance our understanding of lavender’s diverse applications based on its distinct developmental processes but also provide insights for the implementation of biological control strategies.

## Materials and methods

### Plants and insects

*L. angustifolia* cultivar ‘Jingxun 2’ was collected from the National Germplasm Bank of Aromatic Plants, located at the Institute of Botany, Chinese Academy of Sciences, at different developmental stages. The stages were defined as follows: Bud represents FB0, characterized by fully closed and green petals; Blossom denotes the blossom stage, with approximately 60% of the flowers open and the petals displaying a purple colouration (F3); and Fade represents the fading stage, in which the petals had started to shrink and were on the verge of falling off (F5). Each stage has been described in a previous study [19]. *Nicotiana benthamiana* (benth) and *A. thaliana* plants at four weeks of age were cultivated in a controlled greenhouse environment at a temperature of 25 °C, with a photoperiod of 16 h of light and 8 h of darkness and a light intensity of 200 mmol m^− 2^s^− 1^.

The aphids (*Myzus persicae*) and ladybugs (*Harmonia axyridis*) used in the experiments were graciously provided by Prof. Su Wang from the Beijing Academy of Agricultural and Forestry Sciences. The aphids were maintained in a growth chamber measuring 30 × 30 × 25 cm, set at a temperature of 25 °C, and subjected to an L16:D8 photoperiod. The aphids were fed on tobacco plants. Ladybugs that emerged from pupae were kept at a temperature of 25 °C, while the remaining pupae were stored at a temperature range of 10–15 °C.

### Cloning and transcript analyses of the *LaTPS7*, *LaTPS8* and *LaCYP71D582* genes

Total RNA was extracted from the glandular trichomes of both flowers and leaves for further analysis. To obtain RNA, the glandular trichomes were carefully scraped along the base using a needle. For leaves, trichomes at various developmental stages were collected by brushing and then sequentially filtered through meshes with pore sizes of 1.6 mm, 0.15 mm, and finally 0.125 mm to separate glandular trichomes from flowers and leaves. Liquid nitrogen was employed to maintain the integrity of the trichomes during the collection process as described previously [20]. The glandular trichomes were observed under a microscope after their isolation.

Once the RNAs were collected, reverse transcription was conducted using oligo d(T) primers according to the manufacturer’s instructions (TSINGKE, China). Following cDNA synthesis, target genes were amplified utilizing gene-specific primers and Phanta Max Polymerase (Vazyme, China). To determine the expression levels, the relative quantification method was employed, with 18S rRNA serving as the reference gene to calculate the normalized expression levels (2^−△△CT^). Statistical analysis of gene expression levels across different flower developmental stages was performed using least significant difference (LSD) test in SPSS 17.0. The notation ‘abc’ was employed to indicate significant differences. Detailed information regarding the primers used in this study can be found in Table S1.

### Sequence analysis of the target genes

Multiple sequence alignment was performed on ESPript (http://espript.ibcp.fr/ESPript/ESPript/) following a previously reported methodology [21]. The primers employed for ORF amplification can be found in Table S1. Phylogenetic trees were generated using the neighbour-joining method in MEGA 7, and the resulting trees were visually enhanced using Interactive Tree of Life (http://itol2.embl.de/) [22]. Additional details regarding the genes utilized in constructing the phylogenetic tree can be found in Tables S2 and S3.

### Subcellular localization of LaTPS7, LaTPS8, and LaCYP71D582 in *N. benthamiana*

The mannopine synthase promoter MAS-driven expression vector super-1300-eGFP was subjected to Hind III digestion in preparation for ligating the target genes using the TreliefTM SoSoo Cloning Kit (TSINGKE, China). Following the protocol outlined by Jin et al. [23], the resulting plasmids were then transformed into *E. coli* for amplification before being transferred into *Agrobacterium tumefaciens* GV3101 for transient transformation in 4-week-old *N. benthamiana* plants. The leaves of *N. benthamiana* were carefully excised, mounted onto slides, and examined using a confocal laser-scanning microscope equipped with a standard filter set (Leica TCS SP5) after 3 days of infiltration. The acquired images were processed using ImageJ software (https://imagej.nih.gov/ij).

### Heterologous expression of TPSs in *E. coli* and in vitro enzymatic assay

The shortened sequences of terpene synthases (TPS) lacking the anticipated signal peptide were inserted into a pDE2 vector containing a recombinant C-terminal and poly-histidine tag using the pDE2 Directional Expression Kit Ver. 2 (TSINGKE, China). The resulting constructs were then transformed into *E. coli* BL21 (DE3), and the recombinant proteins were induced with isopropyl-*β*-D-thiogalactopyranoside (IPTG) at a concentration of 1.0 mM for approximately 8 h. Subsequently, the proteins were purified using a His-Tagged Gravity Column (Merck Millipore). To confirm successful purification, sodium dodecyl sulfate‒polyacrylamide gel electrophoresis (SDS‒PAGE) was performed.

For the in vitro enzymatic assay to assess TPS activity, purified proteins (approximately 20 µL) were mixed with 500 µL of buffer (25 mM HEPES, pH 7.3; 10 mM MgCl_2_; 10% glycerol; 10 mM DTT) and 10 µg of either farnesyl diphosphate (FPP), neryl diphosphate (NPP), or geranyl diphosphate (GPP) (Sigma‒Aldrich). The enzymatic assay protocol described by Chen et al. [24] was followed. After vortexing, the mixtures were incubated at 30 °C for 2 h. Subsequently, 250 mL of hexane was added and vortexed for 1 min. The upper layers were then centrifuged at 1200 g and 4 °C for 30 min, followed by transfer to 2 mL glass vials for gas chromatography‒mass spectrometry (GC‒MS) analysis. Heat-inactivated recombinant proteins were used as a negative control in the experimental procedure.

### Transient expression of *LaTPS7*, *LaTPS8*, and *LaCYP71D582* in *N. benthamiana*

Upon reaching an optical density (OD_600_) of 1.0, leaves from 4-week-old *N. benthamiana* plants were selected for infiltration with GV3101 harbouring specific target TPS genes. Concurrently, GV3101 strains carrying TPS or CYP were mixed at a 1:1 ratio for coexpression once the OD_600_ reached 1.0. Following a 5-day incubation period, the infiltrated leaf tissues were carefully excised and assessed under UV light to verify the efficiency of agroinfiltration and ascertain gene expression levels. Subsequently, the leaf samples were rapidly frozen using liquid nitrogen and subsequently pulverized. The extraction of volatiles was achieved by subjecting the leaf powder to vortexing in hexane after maintaining a temperature of -80 °C. The resulting extracts were stored at -20 °C prior to analysis. Gas chromatography‒mass spectrometry (GC‒MS) was employed to characterize and quantify the volatile chemicals present in the samples.

### GC‒MS analysis

GC‒MS analysis was performed using an Agilent 7890B GC system coupled with an Agilent Technologies 7000 C Inert XL Mass Selective Detector equipped with an HP-5MS UI column (30 m × 0.25 mm × 0.25 μm; Agilent Technologies). The injection method employed was splitless. The injector temperature was maintained at 250 °C throughout the analysis. The temperature program consisted of an initial hold at 40 °C for 3 min, followed by a linear increase to 130 °C at a rate of 10 °C·min^− 1^, then to 250 °C at a rate of 50 °C·min^− 1^, which was held for 10 min. Helium gas was employed as the carrier gas at a flow rate of 1 mL·min^− 1^.

The ionization energy used was 70 eV, while the electronic impact ion source temperature, quadrupole temperature, and mass range were set at 200 °C, 150 °C, and 35–550 u, respectively. Product identification was achieved by comparing the retention time and electron ionization mass spectra of the detected compounds with those available in the Mass Spectral Library of Agilent.

### Determination of promoter activity in *A. thaliana* and gene expression under methyl jasmonate (MeJA) treatment

Fusion primer and nested integrated PCR (FPNI-PCR) was employed to extract a 1.5-kb region of the promoter for each gene from genomic DNA using gene-specific primers and arbitrary primers, following the PCR conditions described by Wang et al. [25]. The obtained sequences were analysed using the PlantCARE online tool [26] and subsequently inserted into the pCXGUS-P vector, which was digested with BamHI. The pCXGUS-P vector contains a cloning cassette upstream of the GUS gene (uidA). After validation through sequencing, the resulting constructs were introduced into *Agrobacterium* GV3101 and used for floral dip transformation of *A. thaliana*, following the method described by Clough et al. [27]. Transformed lines were selected on MS plates containing 50 µg·mL^− 1^ hygromycin and further confirmed by PCR analysis. GUS staining, based on the technique described by Jefferson et al. [28] using 5-bromo-4-chloro-3-indolyl-*β*-D-glucuronide (X-Gluc), was performed.

Furthermore, at 8:00 AM, lavender plants at the flowering stage were treated with 8 mM methyl jasmonate (MeJA) to stimulate gene expression according to a previous study [20]. The control group received a MeJA-deficient buffer. Each plant was treated with 10 mL of the buffer until the leaves were wet, and after 12 h, the plant materials were harvested and stored in liquid nitrogen as described previously [20]. Using the aforementioned approach, the relative expression levels of the target genes in the glandular trichomes of lavender flowers were examined using quantitative real-time PCR (qRT‒PCR). The significance of *LaTPS7* expression under MeJA treatment was determined using Student’s *t* test in SPSS 17.0.

### Insect behaviour

A Y-tube olfactometer test was conducted to assess the impact of volatiles on the behaviour of aphids (*M. persicae*) and ladybugs (*H. axyridis*) following a fasting period of 3 h. The volatile compounds limonene and carveol were dissolved in paraffin oil to generate different concentrations. The olfactometer was operated at a flow rate of 300 mL·min^− 1^. Insects were categorized as responders if they entered more than half the length of the olfactory arms and remained there for at least 10 s. If the insects did not show a preference for either arm after 5 min, they were classified as having made no choice. After testing 10 aphids or ladybugs, fresh disks were replaced. The significance of the insect choices was examined using the Chi-square test in SPSS 17.0. Each insect was tested only once. To eliminate any residual odour sources, the olfactometer was cleaned with 95% ethanol after each test, followed by washing with distilled water and drying in an oven in preparation for the next sample test. During the measurement process, a fluorescent lamp positioned approximately 15 cm above the front of the Y-shaped tube provided uniform illumination, ensuring consistent light intensity in both arms. The χ^2^-analysis method was employed to evaluate if the insect selection between the treated odour sources followed a theoretical distribution with an h0 of 50: 50 under different criteria, and χ^2^ values were calculated accordingly.

Furthermore, a leaf disk bioassay was conducted following the protocol described by Picard et al [37]. Transformed *N. benthamiana* leaves were punched into 1 cm diameter disks, which were then placed on filter paper inside Petri dishes. Starved aphids were introduced into the Petri dishes, and the number of insects on control and treatment disks was recorded for a duration of 1 hour. A total of 90 aphids were included in the study. The influence of volatiles on ladybugs, also starved for 3 hours, was assessed using a Y-Tube olfactometer. A total of 60 ladybugs were tested. Ladybugs that entered more than half of the arm length of the olfactometer and remained for at least 10 seconds were considered responders. After testing 10 aphids or ladybugs, fresh disks were provided. Subsequent statistical analysis was conducted to analyze the obtained data. The statistical analysis for the aphids was performed using General Linear Model-Univariate Analysis in SPSS 17.0. The model was selected as ‘type III’, and a dual comparative test employing the method of ‘Tukey s-b’ was utilized with a significance level of p = 0.05. For the ladybugs, the statistical analysis involved the use of a χ^2^ test, also conducted in SPSS 17.0.

## Results

### Candidate genes from *L. angustifolia* and construction of a phylogenetic tree

The transcriptomes from flowers at different developmental stages were analysed to investigate gene expression patterns in a previous study [19]. Based on the transcriptome data, TPS (TRINITY DN51614 c1 g5) and CYP (TRINITY DN22690 c0 g1) were selected for further analysis due to their high expression levels in the bud and coexpression pattern (Figure S1). Two TPS genes were identified using the same primer pairs, yielding sequences of 1803 and 1800 bp, and were designated *LaTPS7* and *LaTPS8*, respectively, after validation through sequencing. The nucleotide sequences of *LaTPS7* and *LaTPS8* shared identical regions of 33 bp at the N-terminus and 23 bp at the C-terminus (Figure S2).

**Fig. 1:**
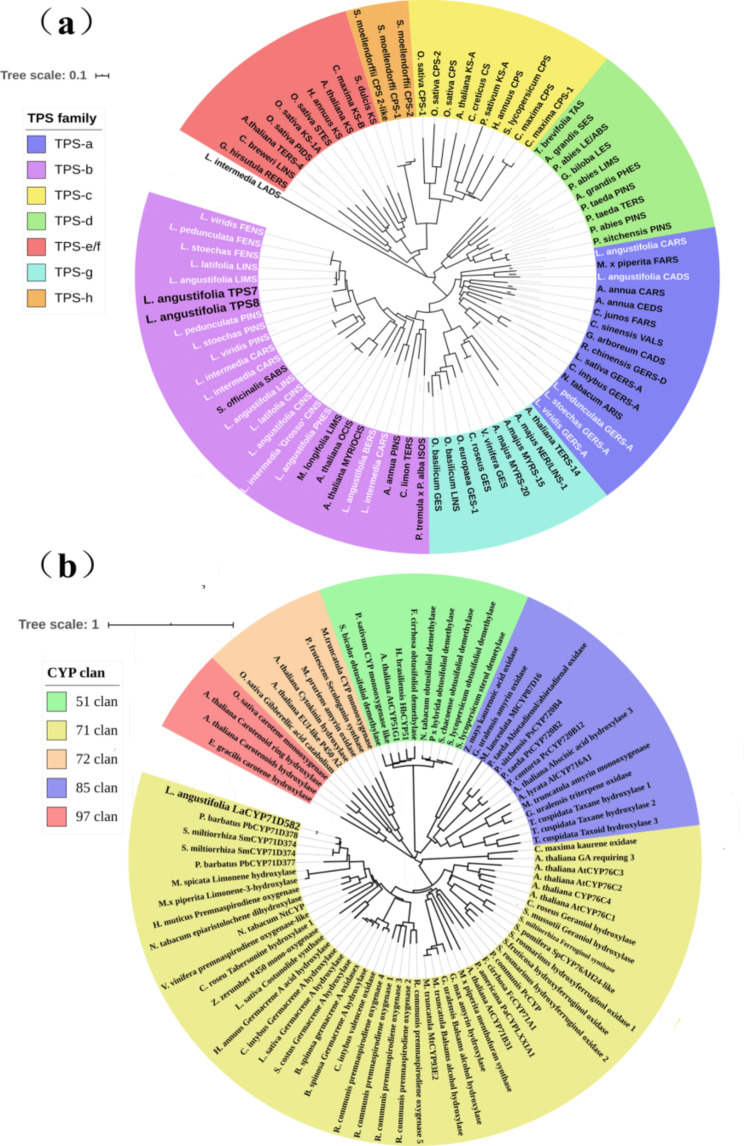
Phylogenetic tree of *LaTPS7*, *LaTPS8* and *LaCYP71D582*. (**a**) The candidate TPSs genes were grouped into TPS-b (purple), which is denoted by bold fonts, and the genes in white font (purple and blue) are all lavender terpene synthase genes that have been published. The monoterpene synthases are clustered into TPS-b (purple), including *LaTPS7* and *LaTPS8.* While the sesquiterpene synthases are clustered into TPS-a (blue). (**b**) According to Nelson and Werck-Reichhart [17], the target gene *LaCYP71D582* was clustered into the CYP71 clan (yellow), which was denoted by bold fonts; CYPs are selected from five clans relative to terpene metabolism. Table S2 for TPSs and Table S3 for CYPs contain the sequences utilized to build the tree. The tree was drawn in MEGA 7 using the Neighbor-Joining method, and then plotted by the web-based iTOL (https://itol.embl.de)

To explore their relationship with other terpene synthases, neighbour-joining phylogenetic trees were constructed based on the deduced amino acid sequences. The analysis revealed that both *LaTPS7* and *LaTPS8* belonged to the TPS-b subfamily of monoterpene synthases [24] (Fig. 1a). *LaTPS7* exhibited the highest similarity to limonene synthase from *L. angustifolia*, while *LaTPS8* showed the highest similarity to pinene synthase from *L. pedunculata*. Notably, both proteins possessed conserved motifs such as the arginine-tryptophan (RRX_8_W) motif at the N-terminus and the aspartate-rich (DDXXD) and NSE/DTE motifs at the C-terminus, which are involved in the cyclization of terpene precursors such as GPP, NPP, and/or FPP (Figure S3, S4).

The CYP sequence was assigned the name *LaCYP71D582* by the CYP450 nomenclature committee. *LaCYP71D582* displayed high identity with *PbCYP71D378* from *Plectranthus barbatus* and exhibited conserved motifs, including (A/G)GX(D/E)T(T/S), EXXR, and FXXGXRXCXG [29] (Fig. 1b) (Figure S5). Phylogenetic analysis of CYP clans associated with terpene metabolism [17] placed *LaCYP71D582* within the CYP71 clan, which is the largest clan among CYPs (Fig. 1b) [30]. Based on these findings, it is likely that *LaCYP71D582* plays a role in terpene biosynthesis.

### Quantitative RT‒PCR analysis of gene expression during the budding stage

**Fig. 2:**
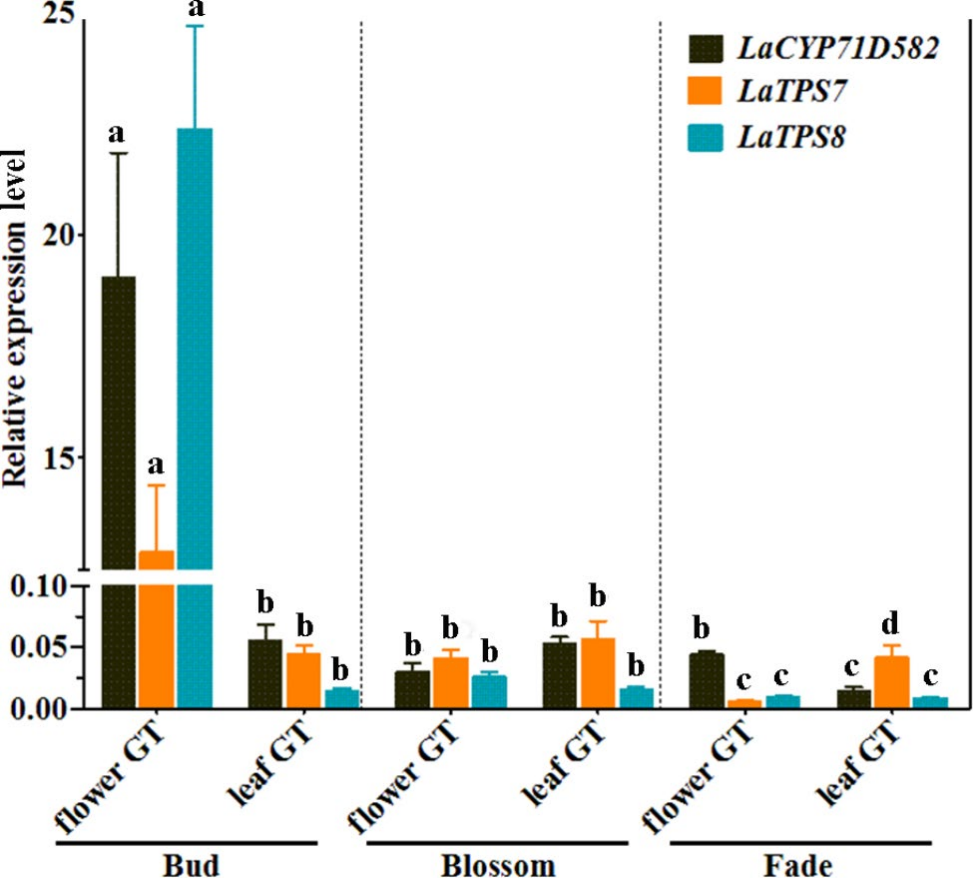
The expression levels of *LaTPS7*, *LaTPS8*, and *LaCYP71D582* in glandular trichomes from various developmental stages were examined by quantitative real-time PCR (qRT-PCR). glandular trichomes were collected from leaves and flowers from bud stage, blossom stag and fade stage. Three genes showed the highest expression level in glandular trichomes of the flower during the bud phase while decreased in blossom and fade stage. The 18 S rRNA genes were used to standardize the transcript levels. The results are the average SE of three replicates

The expression profiles of *LaTPS7*, *LaTPS8*, and *LaCYP71D582* were reassessed using quantitative real-time PCR (qRT‒PCR) in this study. Samples were collected from leaves and flowers at different developmental stages of glandular trichomes, namely, budding, blooming, and fading (Fig. 2). The results revealed that during the budding stage, the expression of *LaTPS7*, *LaTPS8*, and *LaCYP71D582* was highly pronounced in the glandular trichomes of the flowers, whereas negligible expression was observed during other developmental phases. As the flowers transitioned into the blooming and fading stages, the expression levels of *LaTPS7*, *LaTPS8*, and *LaCYP71D582* exhibited a decreasing trend. These findings provide evidence of potential interactions among these genes throughout plant growth (Fig. 2), suggesting that the enzymatic products of TPSs may undergo further modification mediated by CYPs.

### Subcellular localization of LaTPS7, LaTPS8, and LaCYP71D582 via transient expression in *N. benthamiana*

LaTPS7 and LaTPS8 were subjected to signal peptide prediction using Phyre2 software, which indicated the presence of signal peptides. On the other hand, the subcellular localization of LaCYP71D582 was predicted to be in the membrane. To experimentally verify these predictions, ORF fusion vectors containing enhanced green fluorescent protein (eGFP) were constructed for each of these genes. These constructs were then introduced into *N. benthamiana* leaves through *Agrobacterium tumefaciens*-mediated transformation. Subsequently, confocal microscopy was employed to examine the subcellular localization of the proteins of interest.

**Fig. 3:**
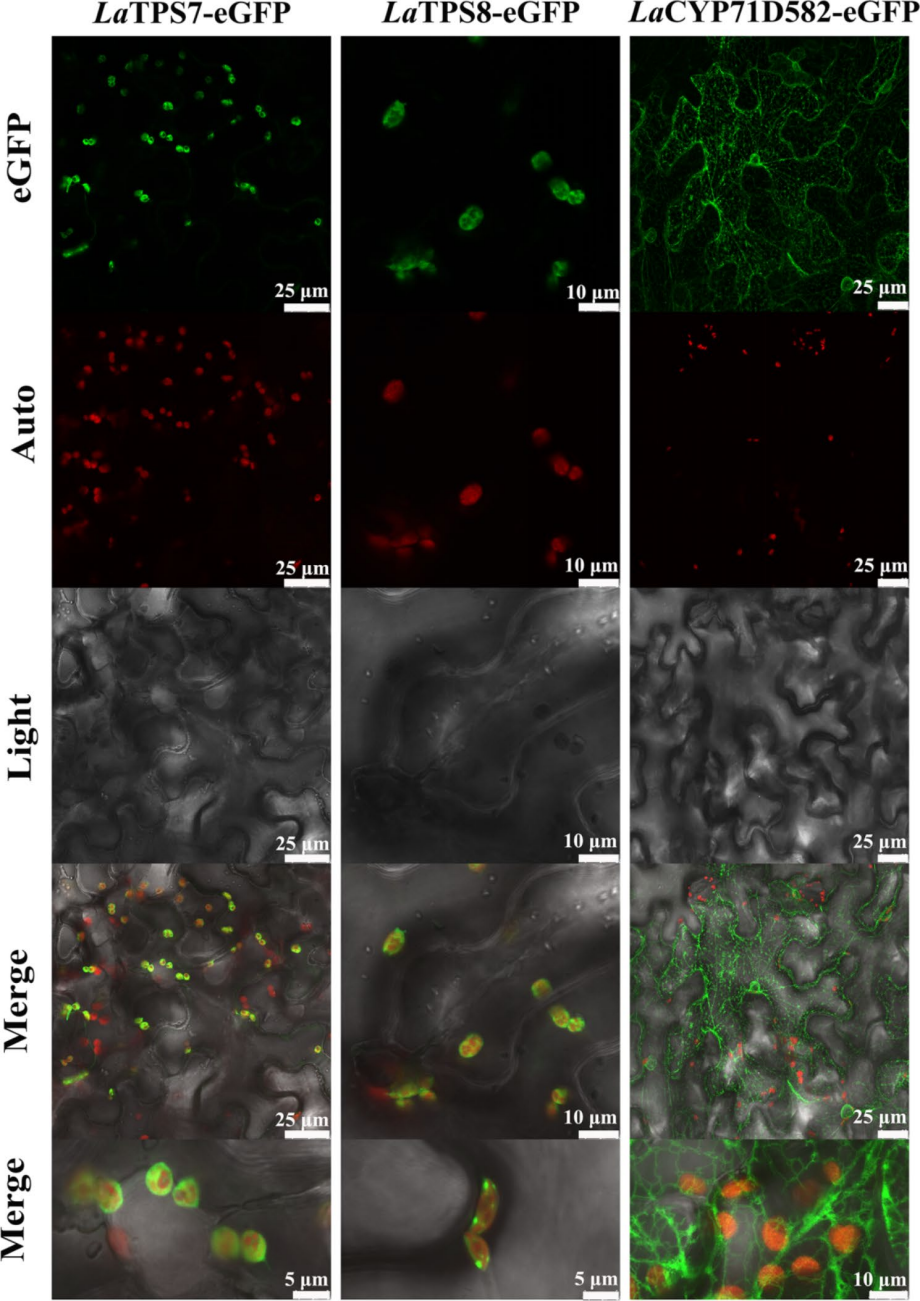
Subcellular localization of LaTPS7, LaTPS8, and LaCYP71D582 in *N. benthamiana*. plant leaves after three days of inoculation were observed under confocal microscopy with 480 nm. LaTPS7 and LaTPS8 were discovered in chloroplasts, particularly, LaTPS8 depicts a scatter distribution, while LaTPS7 depicts a condition in which the chloroplasts are wrapped. Meanwhile, LaCYP71D582 was discovered in the endoplasmic reticulum (ER) contiguous to chloroplasts. Auto, chlorophyll autofluorescence; eGFP, enhanced Green Fluorescent Protein channel image; Light, light microscopy image; Merged, merged image between Auto and eGFP. Post processing of pictures were completed by image J (https://imagej.nih.gov/ij)

The results showed that LaTPS7 and LaTPS8 were localized in the chloroplasts throughout the entire leaf (Fig. 3), confirming their predicted subcellular localization. Conversely, the localization of LaCYP71D582 was observed in the endoplasmic reticulum (ER) (Fig. 3), consistent with the predicted localization.

### Isolation and in vitro enzymatic characteristics of TPSs

To elucidate the functional properties of the proteins under investigation, truncated versions of the TPS proteins lacking signal peptides were generated and fused with a six-His tag to yield recombinant proteins. These recombinant proteins were subsequently expressed and purified in *E. coli* BL21 (DE3). To evaluate the enzymatic activities of the recombinant proteins, they were subjected to assays using GPP (geranyl diphosphate), NPP (neryl diphosphate), or FPP (farnesyl diphosphate) as substrates.

**Fig. 4:**
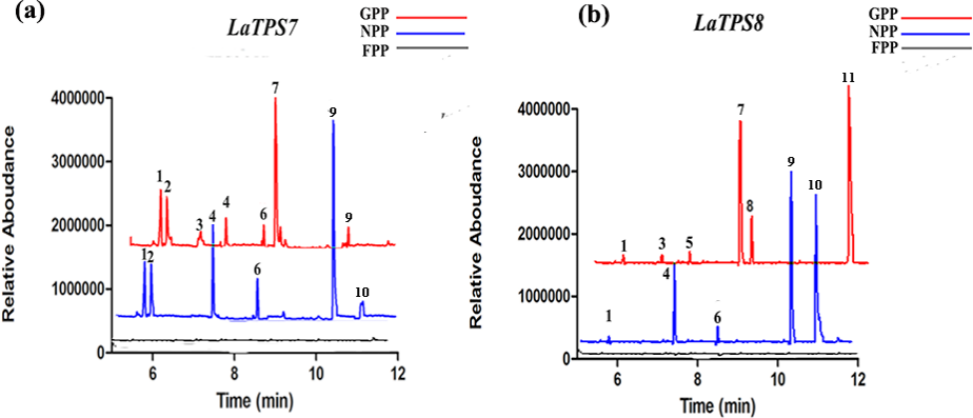
In vitro enzymatic assays of LaTPS7 and LaTPS8. Products produced by LaTPS7 (**a**) and LaTPS8 (**b**) from GPP, NPP and FPP. Compounds are marked with number: (1) *α*-Pinene, (2) Camphene, (3) Myrcene, (4) Limonene, (5) Sylvestrene, (6) Terpinolene, (7) Linalool, (8) Fenchol, (9) Terpineol, (10) Nerol, (11) Geraniol. For each enzymatic catalysis assay, the chromatography was acquired by importing the.csv file into GraphPad. To avoid any ambiguity caused by data overlap, the GPP results (red) were slightly shifted back and thus staggered with the NPP results (blue)

Upon analysis, it was observed that LaTPS7 exhibited catalytic activity towards GPP, leading to the production of seven different monoterpenes, namely, α-pinene, camphene, myrcene, limonene, terpinolene, linalool, and terpineol. Furthermore, when NPP was employed as a substrate, LaTPS7 catalysed the synthesis of α-pinene, camphene, limonene, terpinolene, terpineol, and nerol (Fig. 4a).

On the other hand, LaTPS8 generated α-pinene, myrcene, sylvestrene, linalool, fenchol, and geraniol from GPP and α-pinene, limonene, terpinolene, terpineol, and nerol from NPP (Fig. 4b). Notably, when FPP was utilized as a substrate for either protein, no discernible products were observed. It is important to note that no novel compounds were detected in the control group (data not shown). These results provide evidence that both LaTPS7 and LaTPS8 function as monoterpene synthases, producing distinct volatile compounds from identical substrate molecules.

### Functional characterization of TPSs and CYP in vivo

**Fig. 5:**
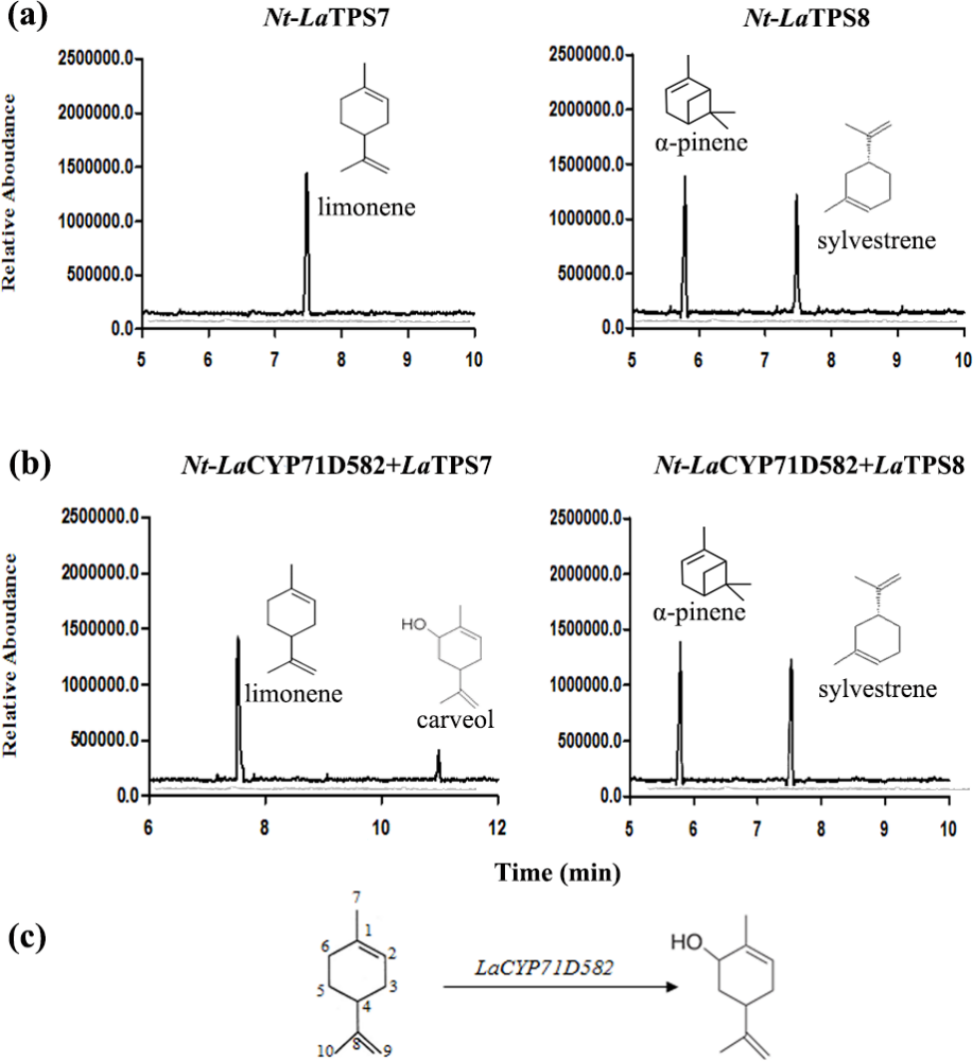
In vivo enzymatic activity in *N. benthamiana*. (**a**) Volatiles produced in *N.benthamiana* leaves over-expressing LaTPS7 or LaTPS8 respectively, showing that LaTPS7 only produced limonene and LaTPS8 produced α-pinene and sylvestrene. (**b**) Volatiles produced in the plant leaves co-expressing LaCYP71D582-LaTPS7 or LaCYP71D582-LaTPS8. It indicated that limonene can be converted into carveol by LaCYP71D582. (**c**) Limonene was hydroxylated at C6 by LaCYP71D582 to form carveol. The gray trace in (**a**) and (**b**) indicate the control extract *N.benthamiana* leaves transformed with empty vector. The chromatography was obtained by GraphPad with the same method as demonstrated in Fig. 4

To validate the functional roles of LaTPS7, LaTPS8, and LaCYP71D582 in plants, transient transfection experiments were conducted in tobacco leaves. Additionally, to investigate their in vivo functions, coexpression experiments were performed involving LaCYP71D582 along with either LaTPS7 or LaTPS8. Notably, in *N. benthamiana*, the expression of *LaTPS7* alone led to the production of limonene, while *LaTPS8* was found to generate *α*-pinene and sylvestrene, as depicted in Fig. 5a.

Moreover, the presence of a newly detected product, carveol, was observed exclusively in the assay involving *N. benthamiana* coexpressing LaTPS7 and LaCYP71D582, while no carveol production was observed in the assay involving LaTPS8 and LaCYP71D582, as depicted in Fig. 5b. These results suggest that LaCYP71D582 potentially plays a role in hydroxylating limonene at the C6 position, resulting in the production of carveol (Fig. 5c).

### The spatial-temporal expression pattern of *LaTPS7* and *LaCYP71D582*

The specific expression patterns of *LaCYP71D582* and *LaTPS7*, which are involved in sequential catalysis, were investigated in this study. To analyse their expression profiles, we employed FPNI-PCR to obtain the promoters of *LaTPS7* and *LaCYP71D582*, referred to as *Pro-LaTPS7* and *Pro-LaCYP71D582*, respectively, resulting in DNA fragments of 1299 and 1434 bp in size. Analysis of these promoters revealed the presence of predicted regulatory elements along with the basic promoter elements, as determined by PlantCARE (Figure S6, S7).

**Fig. 6:**
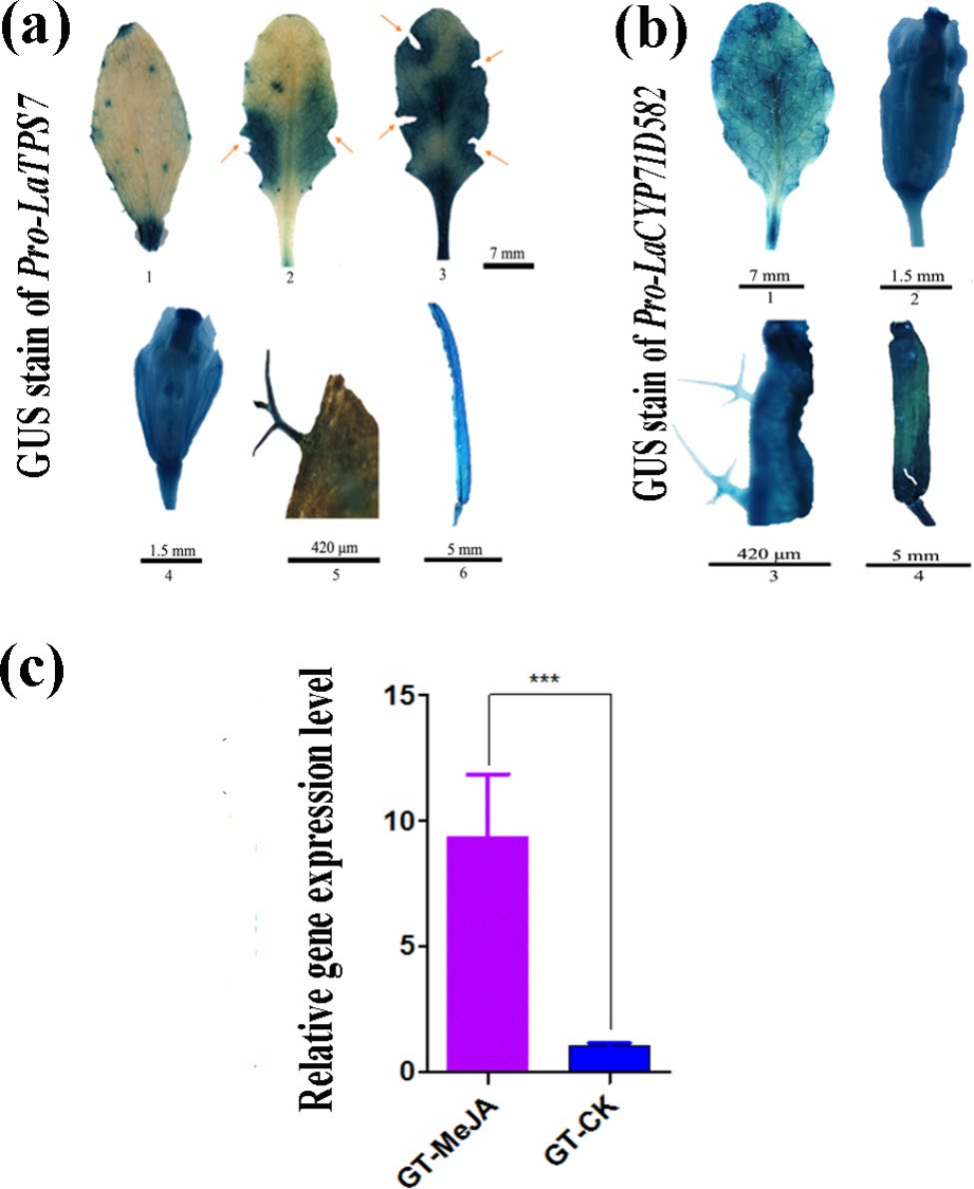
GUS staining and MeJA treatment showing the expression pattern of *LaTPS7* and *LaCYP71D582*. (**a**) Histochemical stain of promoter of *LaTPS7* (*Pro-LaTPS7*). 1-3, leaves; 4, flower; 5, trichome (GT); 6, silique. The red arrows shows the wounding sites. It indicated that the wound is able to stimulate *LaTPS7* expression level; (**b**) Histochemical stain of promoter of *LaTPS8* (*Pro*-*LaCYP71D582*). 1, leaf; 2, flower; 3, trichome (GT); 4, silique. (**c**) The expression level of *LaTPS7* was confirmed by applying 8 mM 10mL MeJA treatment at the blossom stage, when limonene declined. The samples were collected after 12 h. MeJA was dissolved in water with 1% ethanol and 0.1% Tween 20. Asterisks indicate a significant difference of expression level by utilizing Student’s t-test, *** P < 0.001

Subsequently, we transformed promoter‒GUS fusion vectors into *A. thaliana* to examine the expression patterns of *LaTPS7* and *LaCYP71D582*. The GUS reporter gene driven by the *Pro-LaTPS7* promoter showed strong expression in the flowers, siliques, trichomes, and leaves, with notable induction in response to mechanical wounding (Fig. 6a). Notably, the expression of *LaTPS7* in plants was comparable to the response elicited by herbivore attacks. Conversely, *Pro-LaCYP71D582*-driven GUS expression exhibited a constitutive pattern in the flowers, siliques, trichomes, and leaves without being influenced by mechanical injury (Fig. 6b).

Furthermore, to simulate insect infestation, we treated lavender plants at the flowering stage with methyl jasmonate (MeJA), a phytohormone that is recognized to be induced by herbivore attacks and is commonly employed to simulate insect attacks on plants [31–33]. Interestingly, we observed that the expression level of *LaTPS7*, which was expressed at low levels during the blossom stage (Fig. 2), was consistently nearly 10 times higher in glandular trichomes of MeJA-treated flowers than in control glandular trichomes (Fig. 6c). The findings of this study suggested that the expression of *LaTPS7* can be induced by the methyl jasmonate (MeJA) signal. This molecular mechanism is likely to play a significant role in the plant’s defence response.

### Limonene and carveol repel aphids and attract ladybugs to protect plants

Previous studies have indicated that limonene is repellent to most herbivores, including aphids [19]. However, the effects of limonene and its derivatives on aphid-ladybug interactions remain largely unexplored. In this study, researchers employed a Y-tube olfactometer to investigate the responses of aphids and ladybugs to limonene and carveol, aiming to elucidate the potential relationship between volatile chemicals, aphids, and ladybugs.

**Fig. 7:**
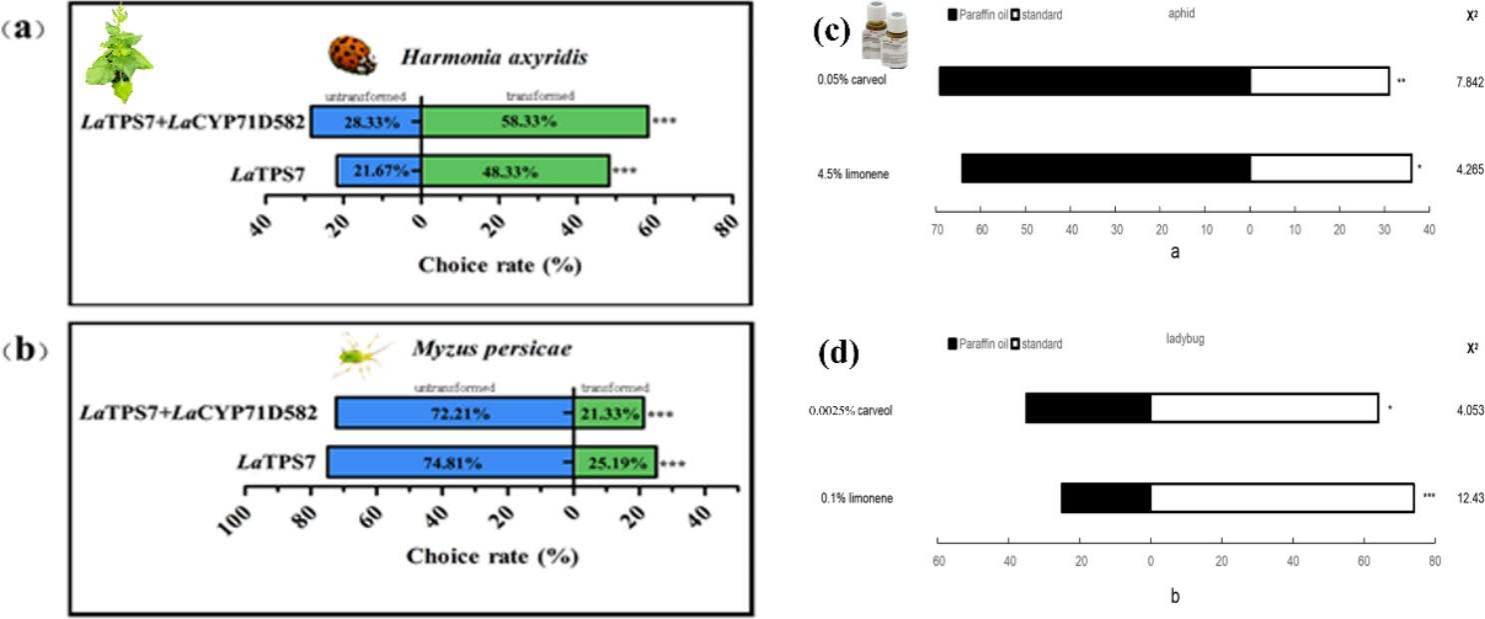
Behavioral responses of aphids and ladybugs toward overexpressed plants and standards. Over 70 aphids and ladybugs were tested in total for each odor. Asterisks indicate a significant difference of choice towards different odor source using Chi-Squared Test. *P < 0.05; **P < 0.01; *** P < 0.001). Y-Tube olfactorometer test was involved and the χ^2^-analysis method was utilized to see if insect selection between the χ^2^ treated odor sources followed a theoretical distribution with a h0 of 50:50 under various criteria, and χ^2^ values were calculated. Statistics for aphids in leaf disk bioassay was General Linear Model-Univariate Analysis

Consistent with expectations, the results showed that out of 47 tested aphids, 27 preferred paraffin oil over limonene at a concentration of 4.5%. In contrast, 48 aphids avoided the 0.05% carveol, while 22 aphids were attracted to it. Based on these observations, the repellent effectiveness of limonene and carveol against aphids was estimated to be approximately 65%. Surprisingly, ladybugs exhibited contrasting behaviour, as 55 individuals were attracted to limonene at a concentration of 0.1%, while only 19 chose paraffin oil. Similarly, 45 ladybugs were attracted to the 0.025% carveol, whereas 25 preferred the paraffin oil. The attraction rates for limonene and carveol were approximately 60% and 70%, respectively (Fig. 7c, d).

Benth leaves transfected with LaTPS7, and leaves co-transfected with LaTPS7 and LaCYP71D582, exhibited a repellent effect on aphids, with an approximate rate of 70% (Fig. 7b). In comparison, leaves with an empty vector displayed a repellent rate of approximately 20%. Conversely, tobacco leaves expressing LaTPS7 attracted ladybugs at a rate of 48.33%, while leaves co-expressing LaTPS7 and LaCYP71D582 attracted ladybugs at a slightly higher rate of 58.33% (Fig. 7a). It is worth noting that the percentages do not sum up to 100% due to the presence of some insects that did not choose either side. These findings suggest that the combination of limonene and carveol is more attractive to ladybugs than limonene alone.

These findings shed light on the potent anti-aphid impact of limonene and carveol, as they effectively inhibited aphid attacks on the plant. The results indicated a complex and contrasting response of aphids and ladybugs to these volatile compounds, highlighting the intricate interplay between the plant, herbivores, and natural enemies in the context of plant defence mechanisms.

## Discussion

During the early developmental stages, in contrast to the mature stages, plants allocate resources towards the production of a diverse array of volatiles due to the vulnerability of juvenile tissues, such as young leaves and blossoms, to herbivore attacks [7]. This phenomenon is supported by gene expression profiles obtained during the flower development phase (Figure S1), which highlight the importance of various volatile compounds in plant defence mechanisms during the early stages. Similar observations have been reported in other plant species, including *A. thaliana* and *Zanthoxylum piperitum* [5, 34]. Balancing growth and defence during this period poses a challenge for plants, as both processes require substantial energy [7]. Consequently, plants have evolved multiple strategies to ensure their survival and growth within limited spatial constraints.

Our analysis revealed that limonene and ocimene are dominant compounds that potentially confer protection against herbivores during the budding stage [19]. Conversely, volatiles associated with attracting pollinators during anthesis, such as linalool, linalyl acetate, and lavandulyl acetate, were prominent. These findings are consistent with the results reported by Pokajewicz et al. [35]. However, an interesting discrepancy was noted, as terpinen-4-ol emerged as a major constituent during the blossom stage. This discrepancy could potentially be attributed to genetic variation. Notably, both limonene and carveol exhibited increased levels in the new cultivars *L.* x *intermedia* and *L. angustifolia* [35], suggesting a correlation between these two compounds. Notably, compounds such as limonene, ocimene, and carveol may not meet the normative content requirements. However, considering the diverse array of volatiles present in lavender, further exploration of the functional roles of different compounds, particularly their physiological and ecological significance, holds great promise for expanding the value and application of lavender.

Plants employ volatile chemicals to establish a tritrophic interaction involving plants, herbivores, and carnivores, thereby providing a natural biological control mechanism and minimizing the need for insecticide use, which contributes to a sustainable agricultural system [36].

Our research findings indicate that limonene and carveol exhibit repellent effects on aphids while attracting ladybugs. Similar observations have been made in soybeans (*Glycine max*) and mint [4, 6]. Furthermore, limonene has been found to attract predatory mites, such as *Phytoseiulus persimilis* and *Neoseiulus californicus* [6], while repelling western flower thrips (*Frankliniella occidentalis*) [37], indicating its potential for resistance against a broad range of herbivores [38]. Carveol, pinene, and limonene may also contribute to the development of plant-based insect repellents. However, insects have evolved resistance mechanisms and flexibility in response to these defensive compounds. For instance, *Musca domestica* employs endogenous P450 enzymes to convert limonene to carveol and carvone, thus reducing the toxicity of these compounds to flies [39]. This finding highlights the multifunctional nature of the same terpene in different species and provides insights into the ongoing coevolutionary arms race between insects and chemically defended plants.

Terpene synthases (TPS) are pivotal enzymes responsible for the biosynthesis of terpenes and terpenoids in plants. Despite the availability of whole genome sequences in certain species, such as *Gossypium*, the highly conserved nature of TPS sequences and their repetitive actions pose challenges in their annotation and functional assignment [40]. Thus, experimental validation becomes necessary for function identification. *LaTPS7* and *LaTPS8* exhibit significant similarity, with 77.61% of their genes being identical, leading to the generation of highly repetitive products in vitro. Both LaTPS7 and LaTPS8 demonstrate the ability to catalyse GPP and NPP into distinct products, thereby illustrating the promiscuity and multisubstrate properties of TPSs [41]. While most monoterpene synthases, including LaTPS7 and LaTPS8, are localized in plastids, TPSs have also been identified in mitochondria and the cytosol [42–45]. Moreover, recent discoveries have revealed the existence of noncanonical metabolic pathways. For instance, in roses, a novel enzyme called RhNUDX1 (a nudix hydrolase) has been identified, which produces geraniol and operates through a catalytic mechanism distinct from that of known monoterpene synthases [45]. These findings underscore the current gaps in our understanding of terpene metabolism at a network level, including substrate regulation and the influence of environmental cues.

The promoter activity of TPS genes in the regulation of terpene synthesis was examined in this study. *LaTPS7* promoter analysis using PlantCARE revealed the presence of MYC transcription factors (TFs), which provided insights into the observed wounding-induced expression pattern, as confirmed by GUS staining. The application of methyl jasmonate (MeJA) specifically enhanced the expression of *LaTPS7* during the blooming stage of lavender, resembling the expression pattern reported in *Capsicum annuum* [46, 47]. Additionally, investigations on poplar leaves demonstrated that juvenile leaves exhibited a more robust and rapid transcriptome response to simulated caterpillar feeding than adult leaves [48]. This heightened response in juvenile tissue can be attributed to its increased susceptibility to herbivore attacks, with transcription factors such as NAC, ethylene-insensitive3-like TF, and R2R3-MYB potentially activating fast signal transduction pathways [49, 50]. Although several TFs have been identified in *L.× intermedia* [51], lavender likely possesses additional regulatory elements that modulate terpene production and responsiveness to the environment. However, most of the studies mentioned above primarily focused on the vegetative growth stage; while lavender is considered a shrub with valuable blooms, it is crucial to also investigate the reproductive growth stage. Despite using *A. thaliana*, a species lacking glandular trichomes but possessing trichomes, in the promoter test experiment, previous research by Beilstein et al. [52] demonstrated the homology between trichomes and glandular trichomes. Hence, considering the absence of genetic manipulation techniques in lavender, *A. thaliana* serves as a suitable model organism for such investigations.

Cytochrome P450 enzymes (CYPs) play a significant role in plant defence mechanisms. Examples include CYP51 in *Avena* spp., CYP99A2 and CYP99A3 in *Oryza sativa*, and CYP82G1 in *Arabidopsis*, as reported by Qi et al. [53], Shimura et al. [54], and Lee et al. [55], respectively. These CYPs are involved in the metabolism of various terpenoids, including mono-, sesqui-, and di-terpenoids, which are initially synthesized and stored in plastids. Subsequently, these terpenoids undergo further modification by CYPs located in the cytosol or endoplasmic reticulum (ER) membranes. During the early flowering stages, *LaCYP71D582*, the first cloned CYP gene from lavender, contributes to plant defence by converting limonene to carveol. Although some CYPs are known to target plastidial membranes [56, 57], this study revealed that *LaCYP71D582* is localized in the ER (Fig. 3), which is in close proximity to plastids within plant cells. This spatial relationship suggests potential communication between these organelles, and similar observations have been reported by Ginglinger et al. [58]. Sequence analysis revealed that *LaCYP71D582* shares 56.40% and 58.72% similarity with limonene-6-hydroxylase from *M. canadensis* (Accession number: QDF63370.1) and *M. gracilis* (Accession number: AAQ18706.1), respectively. In contrast, the most closely related sequence corresponds to a CYP identified in *P. barbatu*s, which is known to be involved in the biosynthesis of forskolin [59]. These findings imply that relying solely on the sequence similarity of CYP enzymes may be unreliable for determining their function and substrates, as highlighted by the investigation conducted by Baldwin et al. [60].

The presence of the GT1-motif, which is light-responsive, and the LTR sequence, which is responsive to low temperature, in the promoter region of *LaCYP71D582* indicates that this CYP gene possesses elements that enable it to respond to environmental stimuli. This observation suggests that environmental factors could potentially influence the expression of CYP enzymes. However, there is a limited amount of research available regarding the impact of environmental variables on CYPs and the regulation of terpenoid biosynthesis. In plants, CYP enzymes are involved in various catalytic and metabolic processes, including the metabolism of fatty acids, alkanes, and phytoalexins [17]. Therefore, it is crucial to not only investigate CYP-related catalytic activities but also examine how biotic and abiotic factors modulate CYP gene expression.

To date, a total of 30 unique CYP genes have been predicted from the expressed sequence tag (EST) database in *L. angustifolia*, as reported by Lane et al. [61]. This valuable resource provides an opportunity for further functional characterization of CYP genes involved in terpene biosynthesis and facilitates a deeper understanding of terpene metabolism in *L. angustifolia*.

In summary, this study investigated the functional roles of three lavender genes, namely, *LaTPS7*, *LaTPS8*, and *LaCYP71D582*, in relation to terpene production and plant defence mechanisms. Specifically, we focused on their involvement during the budding stage, where these genes play significant roles in both direct and indirect defence of lavender plants. Additionally, we examined the interactions between plants, aphids, and ladybugs, which contribute to a tritrophic relationship crucial for successful lavender propagation. Our findings revealed that *LaTPS7* and *LaCYP71D582* exhibit high expression levels in lavender during the budding stage, enabling them to respond effectively to herbivore attacks by producing limonene and carveol as defence mechanisms. Furthermore, establishing a tritrophic connection is essential for optimal plant reproduction and flowering. Consequently, our research expands the range of lavender varieties that can be utilized, owing to a deeper understanding of the diverse developmental processes involved.

### Electronic supplementary material

Below is the link to the electronic supplementary material.

Supplementary Material 1

Supplementary Material 2

Supplementary Material 3

Supplementary Material 4

## Data Availability

The data generated and material used in the current study are available from the corresponding author upon reasonable request.

## References

[1] Ehrlich PR, Raven PH (1964). Butterflies and plants: a study in coevolution. Evolution.

[2] Huang X, Kou J, Jing W, Han X, Liu D, Ghasemzadeh S (2022). Transcriptomic and metabolomic reprogramming in cotton after *Apolygus lucorum* feeding implicated in enhancing recruitment of the parasitoid *Peristenus spretus*. J Pest Sci.

[3] Francis F, Lognay G, Haubruge E (2004). Olfactory responses to aphid and host plant volatile releases: (E)-beta-farnesene an effective kairomone for the predator *Adalia bipunctata*. J Chem Ecol.

[4] Zhu J, Park KC (2005). Methyl salicylate, a soybean aphid-induced plant volatile attractive to the predator *Coccinella septempunctata*. J Chem Ecol.

[5] Boachon B, Junker RR, Miesch L, Bassard JE, Höfer R, Caillieaudeaux R (2015). CYP76C1 (cytochrome P450)-mediated linalool metabolism and the formation of volatile and soluble linalool oxides in *Arabidopsis* flowers: a strategy for defense against floral antagonists. Plant Cell.

[6] Togashi K, Goto M, Rim H, Hattori S, Ozawa R, Arimura GI (2019). Mint companion plants attract the predatory mite *Phytoseiulus persimilis*. Sci Rep.

[7] Meena RK, Jangra S, Wadhwa Z, Monika, Wati L (2017). Role of plant volatiles in defense and communication.Int. J Curr Microbiol App Sci.

[8] Niederbacher B, Winkler JB, Schnitzler JP (2015). Volatile organic compounds as non-invasive markers for plant phenotyping. J Exp Bot.

[9] Ringer KL, Davis EM, Croteau R (2005). Monoterpene metabolism. Cloning, expression, and characterization of (-)-isopiperitenol/(-)-carveol dehydrogenase of peppermint and spearmint. Plant Physiol.

[10] Teoh KH, Polichuk DR, Reed DW, Nowak G, Covello PS (2006). *Artemisia annua* L. (*Asteraceae*) trichome-specific cDNAs reveal CYP71AV1, a cytochrome P450 with a key role in the biosynthesis of the antimalarial sesquiterpene lactone artemisinin. FEBS Lett.

[11] Guitton Y, Nicolè F, Moja S, Benabdelkader T, Valot N, Legrand S (2010). Lavender inflorescence: a model to study regulation of terpenes synthesis. Plant Signal Behav.

[12] Landmann C, Fink B, Festner M, Dregus M, Engel KH, Schwab W (2007). Cloning and functional characterization of three terpene synthases from lavender (*Lavandula angustifolia*). Arch Biochem Biophys.

[13] Demissie ZA, Cella MA, Sarker LS, Thompson TJ, Rheault MR, Mahmoud SS (2012). Cloning, functional characterization and genomic organization of 1,8-cineole synthases from *Lavandula*. Plant Mol Biol.

[14] Demissie ZA, Sarker LS, Mahmoud SS (2011). Cloning and functional characterization of β-phellandrene synthase from *Lavandula angustifolia*. Planta.

[15] Jullien F, Moja S, Bony A, Legrand S, Petit C, Benabdelkader T (2014). Isolation and functional characterization of a τ-cadinol synthase, a new sesquiterpene synthase from *Lavandula angustifolia*. Plant Mol Biol.

[16] Karunanithi PS, Zerbe P (2019). Terpene synthases as metabolic gatekeepers in the evolution of plant terpenoid chemical diversity. Front Plant Sci.

[17] Nelson D, Werck-Reichhart D (2011). A P450-centric view of plant evolution. Plant J.

[18] Guitton Y, Nicolè F, Moja S, Valot N, Legrand S, Jullien F (2010). Differential accumulation of volatile terpene and terpene synthase mRNAs during lavender (*Lavandula angustifolia* and *L* x *intermedia*) inflorescence development. Physiol Plant.

[19] Li H, Li J, Dong Y, Hao H, Ling Z, Bai H (2019). Time-series transcriptome provides insights into the gene regulation network involved in the volatile terpenoid metabolism during the flower development of lavender. BMC Plant Biol.

[20] Dong Y, Zhang W, Li J, Wang D, Bai H, Li H (2022). The transcription factor LaMYC4 from lavender regulates volatile terpenoid biosynthesis. BMC Plant Biol.

[21] Robert X, Gouet P. Deciphering key features in protein structures with the new ENDscript server. Nucleic Acids Res. 2014;42(Web Server issue):W320–4. 10.1093/nar/gku316 PMC4086106 24753421

[22] Letunic I, Bork P (2007). Interactive tree of life (iTOL): an online tool for phylogenetic tree display and annotation. Bioinformatics.

[23] Jin J, Kim MJ, Dhandapani S, Tjhang JG, Yin JL, Wong L (2015). The floral transcriptome of ylang ylang (*Cananga odorata var*. *Fruticosa*) uncovers biosynthetic pathways for volatile organic compounds and a multifunctional and novel sesquiterpene synthase. J Exp Bot.

[24] Chen F, Tholl D, Bohlmann J, Pichersky E (2011). The family of terpene synthases in plants: a mid-size family of genes for specialized metabolism that is highly diversified throughout the kingdom. Plant J.

[25] Wang Z, Ye S, Li J, Zheng B, Bao M, Ning G (2011). Fusion primer and nested integrated PCR (FPNI-PCR): a new high-efficiency strategy for rapid chromosome walking or flanking sequence cloning. BMC Biotechnol.

[26] Rombauts S, Déhais P, Van Montagu M, Rouzé P (1999). PlantCARE, a plant cis-acting regulatory element database. Nucleic Acids Res.

[27] Clough SJ, Bent AF (1998). Floral dip: a simplified method for agrobacterium-mediated transformation of *Arabidopsis thaliana*. Plant J.

[28] Jefferson RA, Kavanagh TA, Bevan MW (1987). GUS fusions: beta-glucuronidase as a sensitive and versatile gene fusion marker in higher plants. EMBO J.

[29] Pateraki I, Andersen-Ranberg J, Jensen NB, Wubshet SG, Heskes AM, Forman V (2017). Total biosynthesis of the cyclic AMP booster forskolin from *Coleus forskohlii*. Elife.

[30] Nelson DR, Schuler MA, Paquette SM, Werck-Reichhart D, Bak S (2004). Comparative genomics of rice and *Arabidopsis*. Analysis of 727 cytochrome P450 genes and pseudogenes from a monocot and a dicot. Plant Physiol.

[31] Senthil-Nathan S (2019). Effect of methyl jasmonate (MeJA)-induced defenses in rice against the rice leaffolder Cnaphalocrocis medinalis (Guenèe) (*Lepidoptera: Pyralidae*). Pest Manag Sci.

[32] Cao Y, Liu L, Ma K, Wang W, Lv H, Gao M (2022). The jasmonate-induced bHLH gene SlJIG functions in terpene biosynthesis and resistance to insects and fungus. J Integr Plant Biol.

[33] Tonğa A, Şeker K, Çakmak S, Temiz MG, Bayram A (2022). Cotton treatment with methyl jasmonate at different growth stages reduces the population of sucking insect pests and marginally increases their associated predators. Entomol Exp Appl.

[34] Fujita Y, Koeduka T, Aida M, Suzuki H, Iijima Y, Matsui K (2017). Biosynthesis of volatile terpenes that accumulate in the secretory cavities of young leaves of japanese pepper (*Zanthoxylum piperitum*): isolation and functional characterization of monoterpene and sesquiterpene synthase genes. Plant Biotechnol (Tokyo).

[35] Pokajewicz K, Białoń M, Svydenko L, Hudz N, Balwierz R, Marciniak D (2022). Comparative evaluation of the essential oil of the new ukrainian *Lavandula angustifolia* and *Lavandula* x *intermedia* cultivars grown on the same plots. Molecules.

[36] Loreto F, Dicke M, Schnitzler JP, Turlings TC (2014). Plant volatiles and the environment. Plant Cell Environ.

[37] Picard I, Hollingsworth RG, Salmieri S, Lacroix M (2012). Repellency of essential oils to Frankliniella occidentalis (*Thysanoptera: Thripidae*) as affected by type of oil and polymer release. J Econ Entomol.

[38] Grace H, Smith JM, Roberts, Tom W (2018). Pope. Terpene based biopesticides as potential alternatives to synthetic insecticides. Crop Prot.

[39] Rossi YE, Palacios SM (2013). Fumigant toxicity of Citrus sinensis essential oil on *Musca domestica* L. adults in the absence and presence of a P450 inhibitor. Acta Trop.

[40] Huang XZ, Xiao YT, Köllner TG, Jing WX, Kou JF, Chen JY (2018). The terpene synthase gene family in *Gossypium hirsutum* harbors a linalool synthase GhTPS12 implicated in direct defence responses against herbivores. Plant Cell Environ.

[41] Major DT, Weitman M (2012). Electrostatically guided dynamics–the root of fidelity in a promiscuous terpene synthase?. J Am Chem Soc.

[42] Yamaga Y, Nakanishi K, Fukui H, Tabata M (1993). Intracellular localization of p-hydroxybenzoate geranyltransferase, a key enzyme involved in shikonin biosynthesis. Phytochemistry.

[43] Aharoni A, Giri AP, Verstappen FW, Bertea CM, Sevenier R, Sun Z (2004). Gain and loss of fruit flavor compounds produced by wild and cultivated strawberry species. Plant Cell.

[44] Lee S, Chappell J (2008). Biochemical and genomic characterization of terpene synthases in *Magnolia grandiflora*. Plant Physiol.

[45] Magnard JL, Roccia A, Caissard JC, Vergne P, Sun P, Hecquet R (2015). Plant volayiles. Biosynthesis of monoterpene scent compounds in roses. Science.

[46] Wu X, Xu S, Zhao P, Zhang X, Yao X, Sun Y (2019). The Orthotospovirus nonstructural protein NSs suppresses plant MYC-regulated jasmonate signaling leading to enhanced vector attraction and performance. PLoS Pathog.

[47] Wang Q, Reddy VA, Panicker D, Mao HZ, Kumar N, Rajan C (2016). Metabolic engineering of terpene biosynthesis in plants using a trichome-specific transcription factor MsYABBY5 from spearmint (*Mentha spicata*). Plant Biotechnol J.

[48] Philippe RN, Ralph SG, Mansfield SD, Bohlmann J (2010). Transcriptome profiles of hybrid poplar (*Populus trichocarpa × deltoides*) reveal rapid changes in undamaged, systemic sink leaves after simulated feeding by forest tent caterpillar (*Malacosoma disstria*). New Phytol.

[49] Nieuwenhuizen NJ, Chen X, Wang MY, Matich AJ, Perez RL, Allan AC (2015). Natural variation in monoterpene synthesis in kiwifruit: transcriptional regulation of terpene synthases by NAC and ETHYLENE-INSENSITIVE3-like transcription factors. Plant Physiol.

[50] Reddy VA, Wang Q, Dhar N, Kumar N, Venkatesh PN, Rajan C (2017). Spearmint R2R3-MYB transcription factor MsMYB negatively regulates monoterpene production and suppresses the expression of geranyl diphosphate synthase large subunit (MsGPPS.LSU). Plant Biotechnol J.

[51] Sarker LS, Adal AM, Mahmoud SS (2019). Diverse transcription factors control monoterpene synthase expression in lavender (*Lavandula*). Planta.

[52] Beilstein MA, Al-Shehbaz IA, Kellogg EA (2006). *Brassicaceae* phylogeny and trichome evolution. Am J Bot.

[53] Qi X, Bakht S, Qin B, Leggett M, Hemmings A, Mellon F (2006). A different function for a member of an ancient and highly conserved cytochrome P450 family: from essential sterols to plant defense. Proc Natl Acad Sci U S A.

[54] Shimura K, Okada A, Okada K, Jikumaru Y, Ko KW, Toyomasu T (2007). Identification of a biosynthetic gene cluster in rice for momilactones. J Biol Chem.

[55] Lee S, Badieyan S, Bevan DR, Herde M, Gatz C, Tholl D (2010). Herbivore-induced and floral homoterpene volatiles are biosynthesized by a single P450 enzyme (CYP82G1) in *Arabidopsis*. Proc Natl Acad Sci U S A.

[56] Kim JE, Cheng KM, Craft NE, Hamberger B, Douglas CJ (2010). Over-expression of *Arabidopsis thaliana* carotenoid hydroxylases individually and in combination with a beta-carotene ketolase provides insight into in vivo functions. Phytochemistry.

[57] Quinlan RF, Shumskaya M, Bradbury LM, Beltrán J, Ma C, Kennelly EJ, Wurtzel ET (2012). Synergistic interactions between carotene ring hydroxylases drive lutein formation in plant carotenoid biosynthesis. Plant Physiol.

[58] Ginglinger JF, Boachon B, Höfer R, Paetz C, Köllner TG, Miesch L (2013). Gene coexpression analysis reveals complex metabolism of the monoterpene alcohol linalool in *Arabidopsis* flowers. Plant Cell.

[59] Ignea C, Ioannou E, Georgantea P, Trikka FA, Athanasakoglou A, Loupassaki S (2016). Production of the forskolin precursor 11β-hydroxy-manoyl oxide in yeast using surrogate enzymatic activities. Microb Cell Fact.

[60] Baldwin SR, Mohapatra P, Nagalla M, Sindvani R, Amaya D, Dickson HA (2021). Identification and characterization of CYPs induced in the Drosophila antenna by exposure to a plant odorant. Sci Rep.

[61] Lane A, Boecklemann A, Woronuk GN, Sarker L, Mahmoud SS (2010). A genomics resource for investigating regulation of essential oil production in *Lavandula angustifolia*. Planta.

