# Supplementary material for: Terpene produced by coexpression of the TPS and P450 genes from Lavandula angustifolia protects plants from herbivore attacks during budding stages
Source: BMC Plant Biol. 2023 Oct 9;23:477. doi: 10.1186/s12870-023-04490-7 (PMC10561503; doi:10.1186/s12870-023-04490-7)
Supplement: Supplementary file 1 — Supplementary Material 1 [file 12870_2023_4490_MOESM1_ESM.docx]

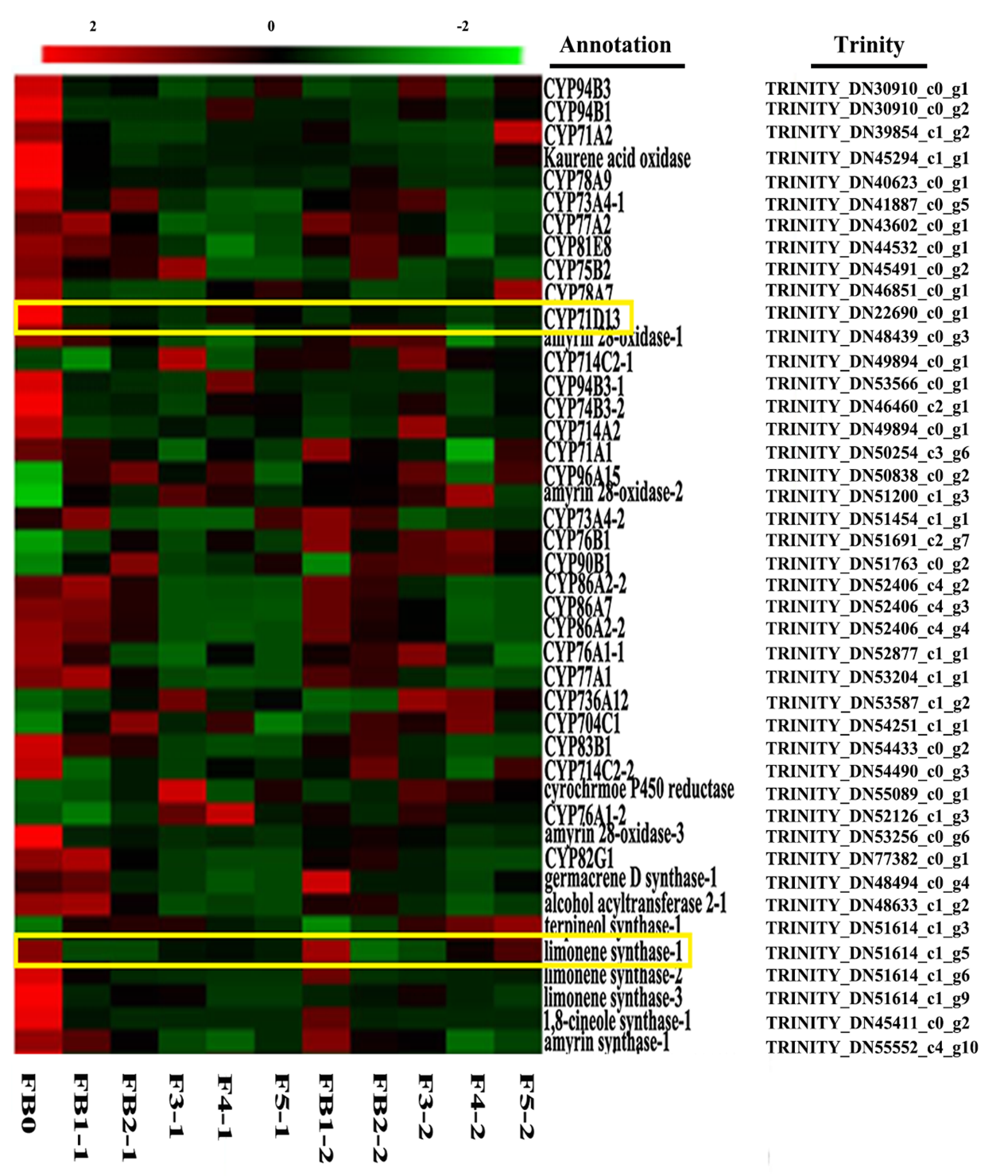


**Figure S1.** Heat map of gene co-expression analysis over various flower development. Yellow boxes highlights the two genes with co-expression tendency annotated as CYP71D13 and limonene synthase-1, respectively. FB0, flower bud. FB1-FB3, blossom. FB4, FB5, fade.


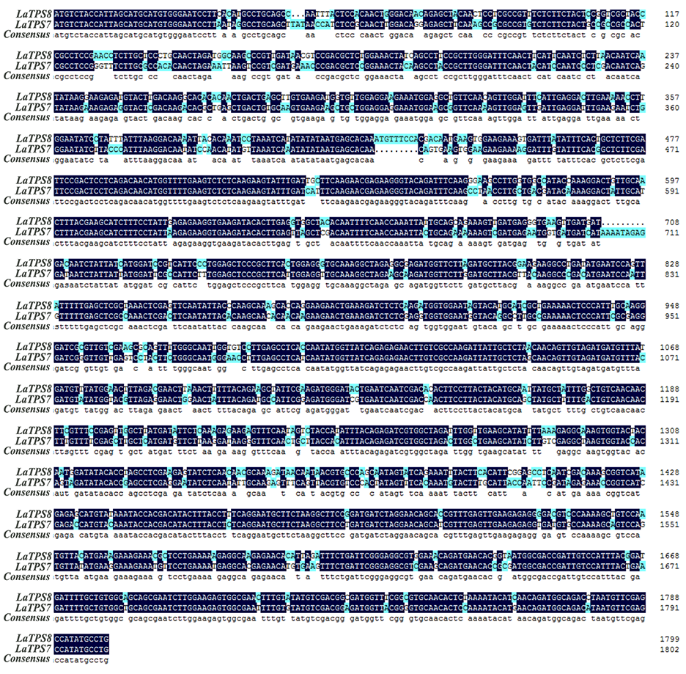


**Figure S2.** The sequence blast of *LaTPS8* and *LaTPS7. LaTPS8* and *LaTPS7* possess identical sequence in their first 33bp and the last 23bp.


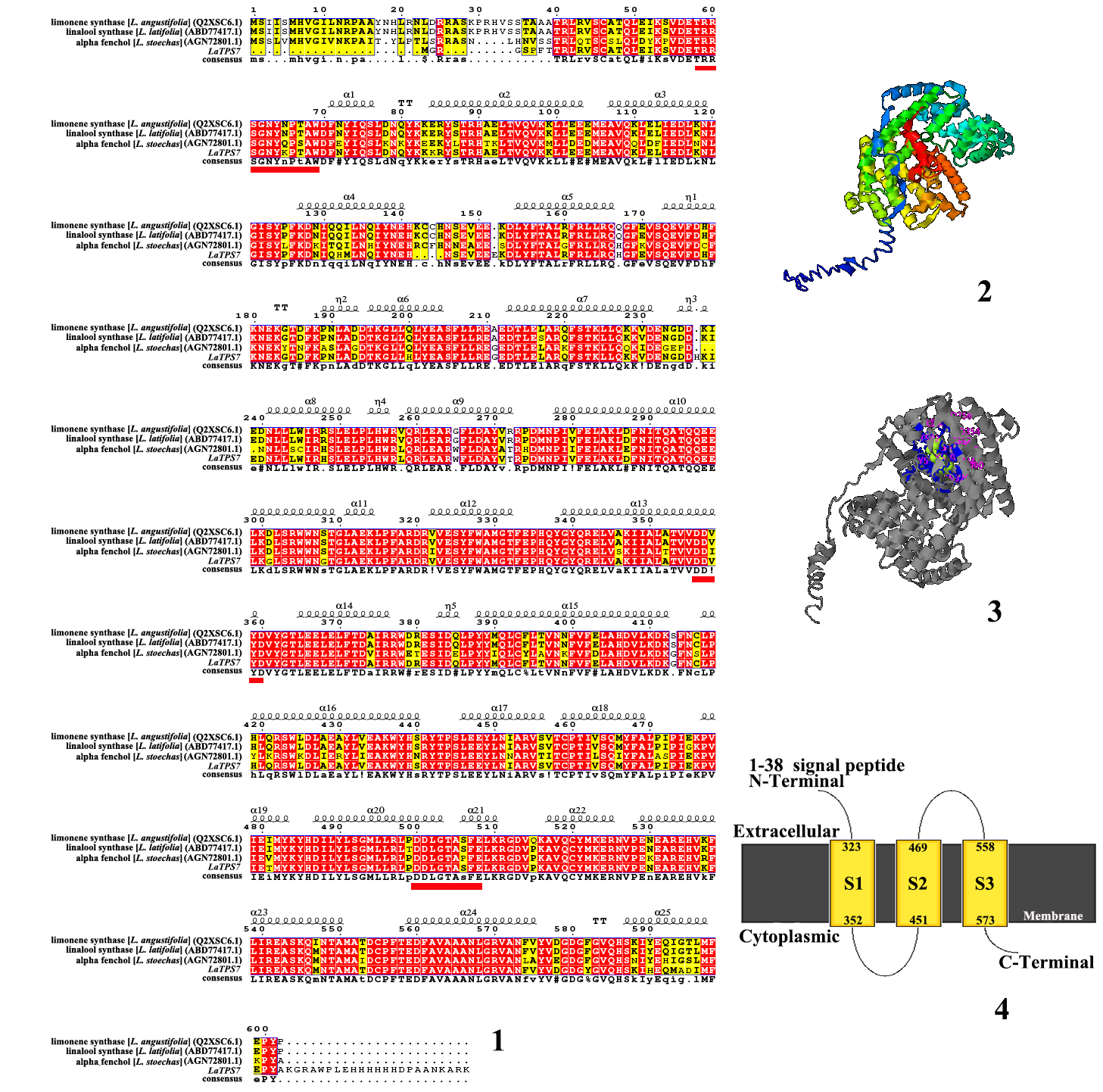


**Figure S3.** Amino acid sequence blast and protein structure of *La*TPS7*.* Amino acid sequence blast. The red lines indicate the conserved domains, including arginine-tryptophan (RRX_8_W), aspartate-rich (DDXXD) and (NSE/DTE) motif.


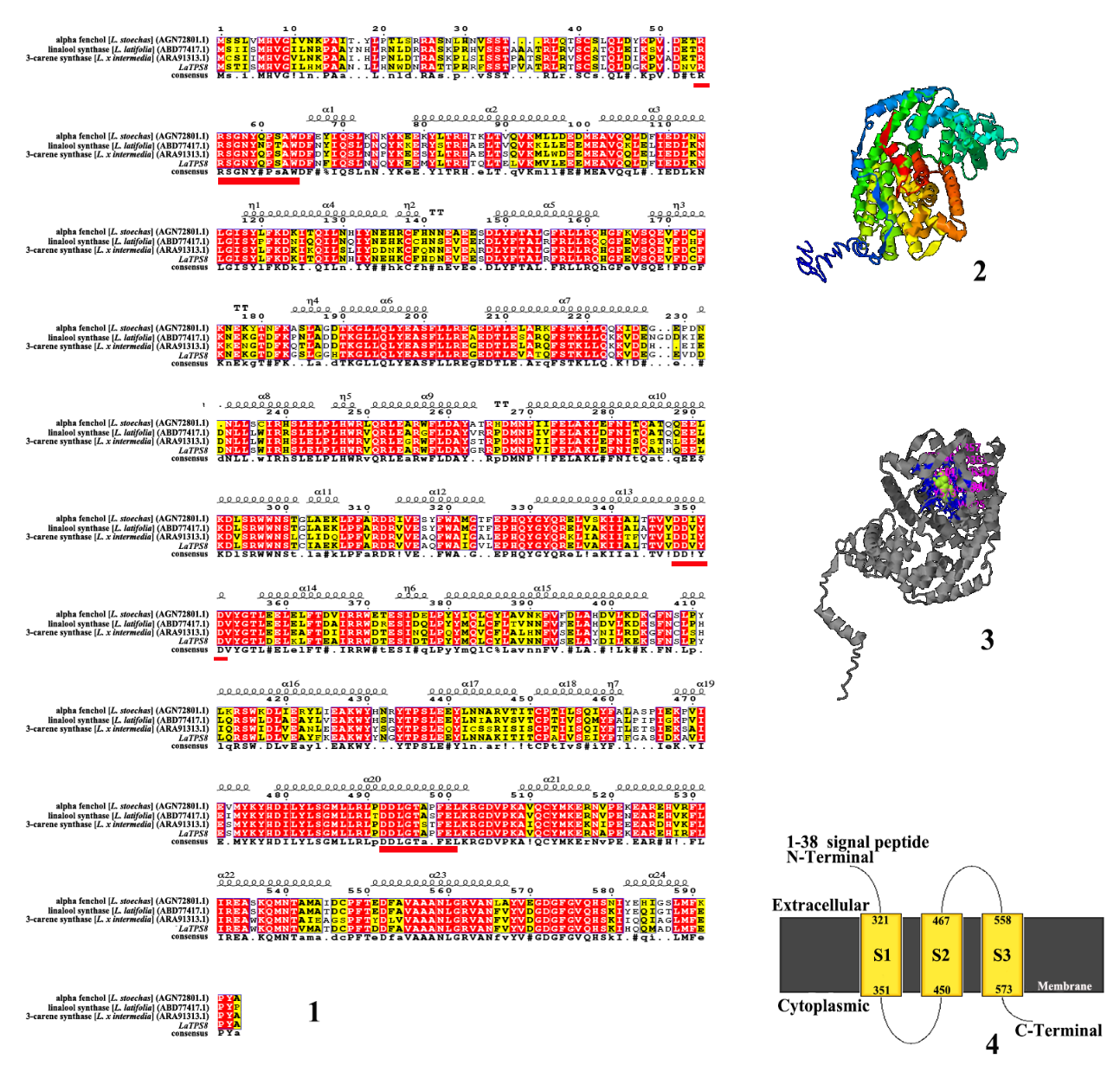


**Figure S4.** Amino acid sequence blast and protein structure of *La*TPS8*.* Amino acid sequence blast. The red lines indicate the conserved domains, including arginine-tryptophan (RRX_8_W), aspartate-rich (DDXXD) and (NSE/DTE) motif.


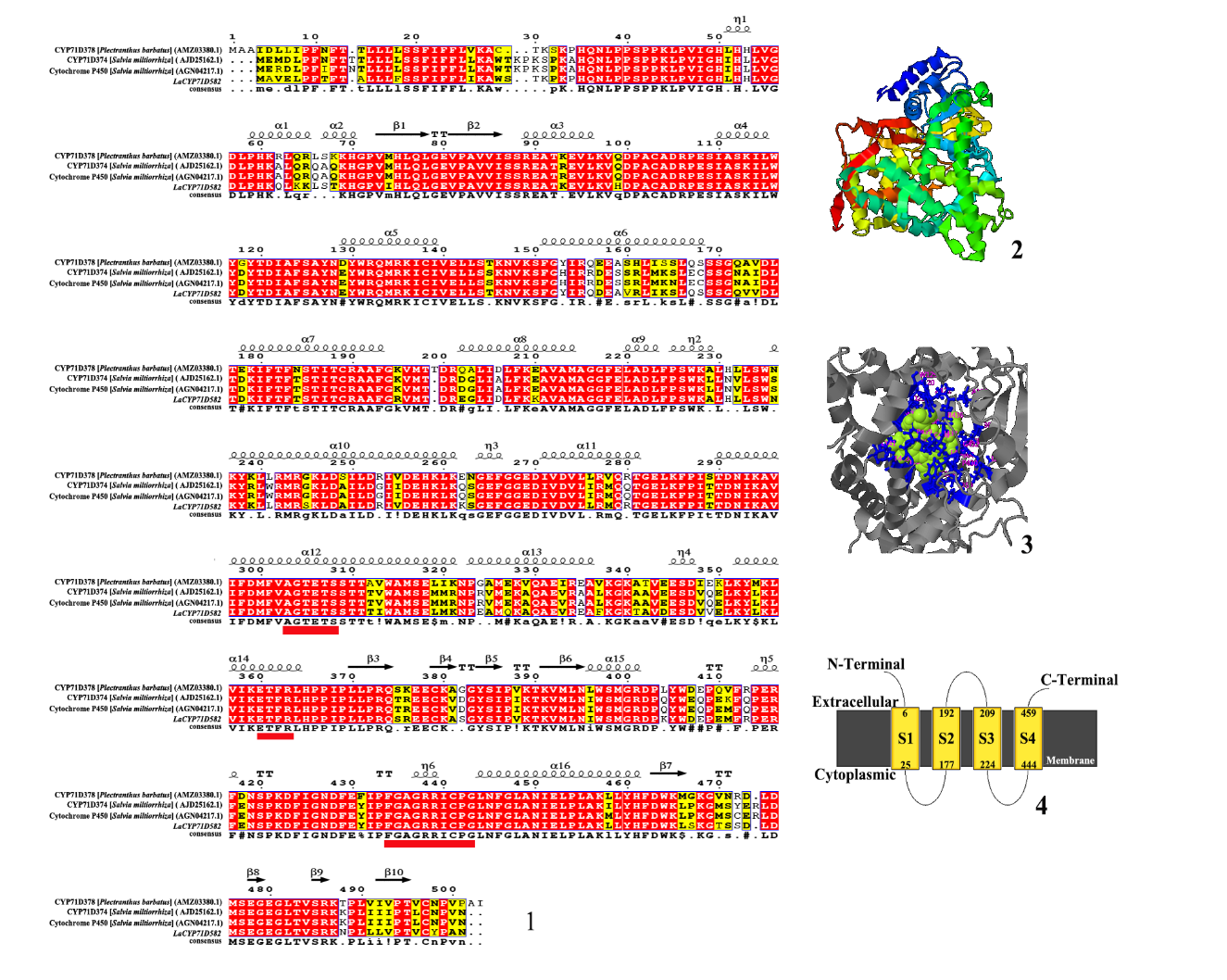


**Figure S5.** Amino acid sequence blast and protein structure of *La*CYP71D582*.* Amino acid sequence blast. The red lines indicate the conserved domains, including (A/G)GX(D/E)T(T/S), EXXR, and FXXGXRXCXG.


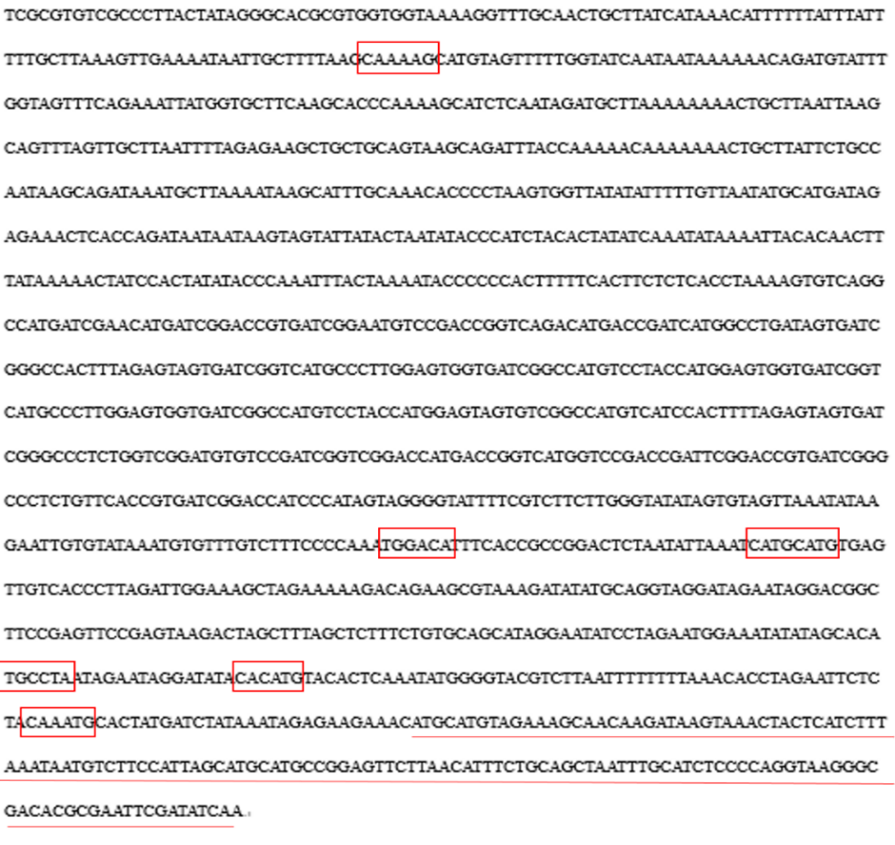


**Figure S6.** Promoter sequence and components of *LaTPS7.* Red boxes indicate MYC motif. Red line, initiation codon of gene.


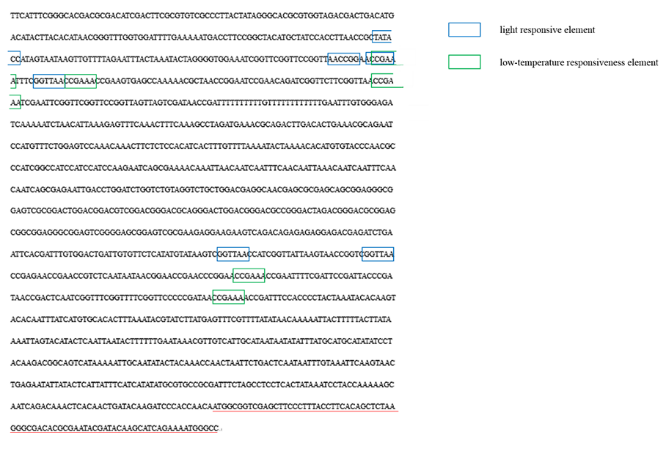


**Figure S7.** Promoter sequence and components of *LaCYP71D582.* Red line, initiation codon of gene. Blue boxes, light responsive element. Green boxes, low-temperature responsive element.


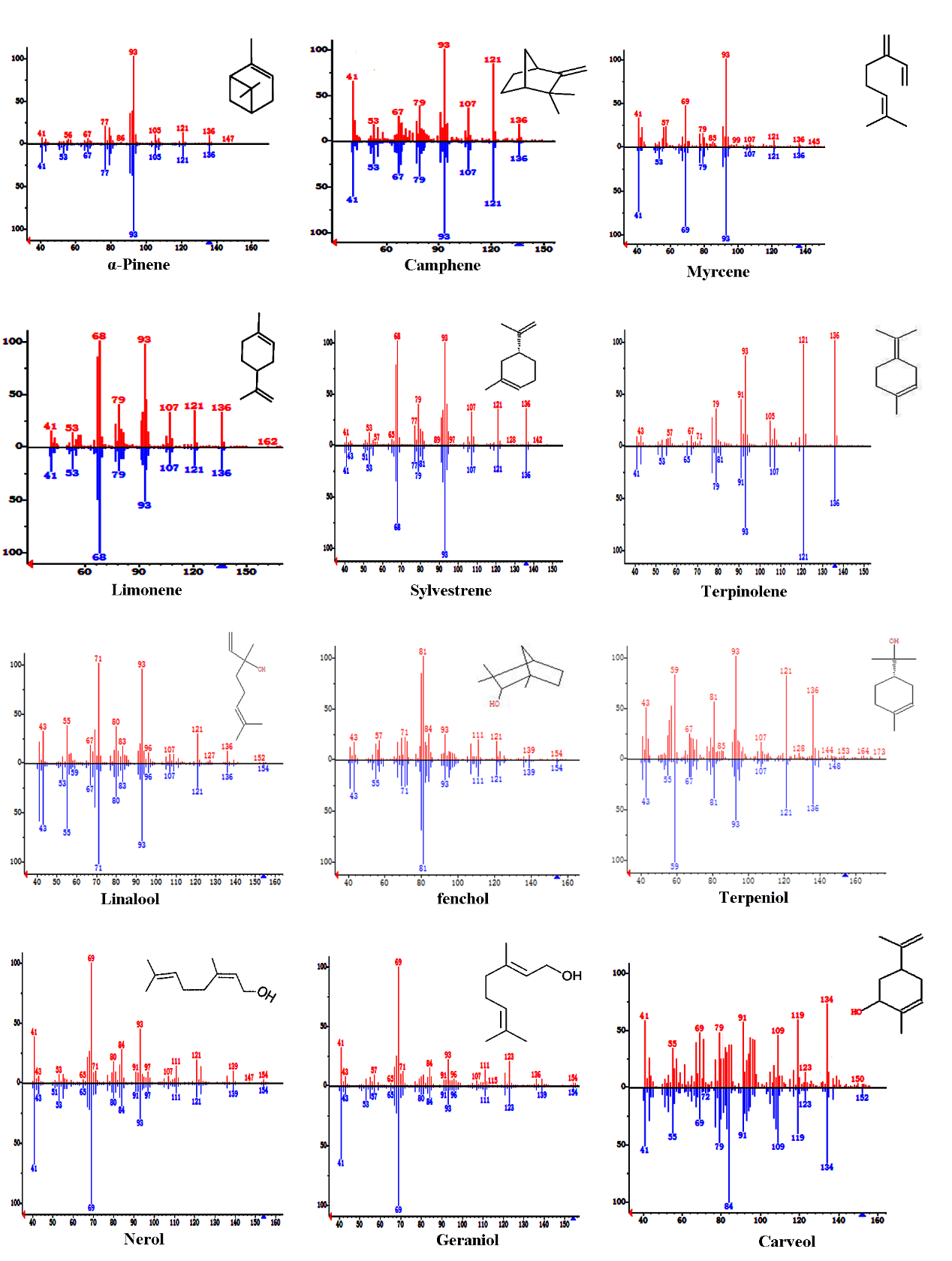


**Figure S8.** Chemical structures and mass spectra of compounds. Mass spectrum of compounds (red), and comparison with mass spectra of authentic standard (blue) form the library of Agilent.
