# Supplementary material for: Terpene produced by coexpression of the TPS and P450 genes from Lavandula angustifolia protects plants from herbivore attacks during budding stages
Source: BMC Plant Biol. 2023 Oct 9;23:477. doi: 10.1186/s12870-023-04490-7 (PMC10561503; doi:10.1186/s12870-023-04490-7)
Supplement: Supplementary file 2 — Supplementary Material 2 [file 12870_2023_4490_MOESM2_ESM.docx]

| Primer | Genes | Sequences |
| --- | --- | --- |
| Clone |  |  |
| 1 | *LaTPS* | F: 5’-ATGTCTACCATTAGCATGCATGTGG-3’ |
|  |  | R: 5’-TCAGGCATATGGCTCGAACATTATG-3’ |
| 2 | *LaCYP* | F: 5’-ATGGCGGTCGAGCTTCCCTT-3’ |
|  |  | R: 5’-TCAGTTAGCCGGGTAGCAGACG-3’ |
| qRT-PCR |  |  |
| 1 | 18S rRNA | F: 5’-AACGACTCTCGGCAACGGATA-3’ |
|  |  | R: 5’-GCGTTCAAAGACTCGATGGT-3’ |
| 2 | *LaTPS7* | F: 5’-GCGCCGTGTCTCTTCTACTG-3’ |
|  |  | R: 5’-TCCCAAGCGGTAGGCTTGTA-3’ |
| 3 | *LaTPS8* | F: 5’-ACTTCACATTCGGAGCCTCA-3’ |
|  |  | R: 5’-AACTCAAACGGTGCTGTTCC-3’ |
| 4 | *LaCYP71D582* | F: 5’-TGGGAATCCTTCACATGCCT-3’ |
|  |  | R: 5’-TTGCCATCTAGTTGCAGGGA-3’ |

Table S1. Primers used in experiment
