# Supplementary material for: Terpene produced by coexpression of the TPS and P450 genes from Lavandula angustifolia protects plants from herbivore attacks during budding stages
Source: BMC Plant Biol. 2023 Oct 9;23:477. doi: 10.1186/s12870-023-04490-7 (PMC10561503; doi:10.1186/s12870-023-04490-7)
Supplement: Supplementary file 3 — Supplementary Material 3 [file 12870_2023_4490_MOESM3_ESM.docx]

**Table S2. Amino acid sequences used for building TPS phylogenetic** **tree**

| TPS fimaly | Species | Protein ID/Accession | Note |  |
| --- | --- | --- | --- | --- |
| TPS-a | *Citrus junos* | AAK54279.1 | (E)-beta-farnesene synthase |  |
|  | *Mentha* x *piperita* | CAH10289.1 | (E)-beta-farnesene synthase |  |
|  | *Lactuca sativa* | XP_023734561.1 | germacrene A synthase |  |
|  | *Artemisia annua* | PWA59867.1 | beta-caryophyllene synthase |  |
|  | *Artemisia annua* | CAC08805.1 | epi-cedrol synthase |  |
|  | *Nicotiana tabacum* | AFJ04408.1 | 5-epi-aristolochene synthase |  |
|  | *Gossypium arboreum* | KHG27433.1 | (+)-delta-cadinene synthase |  |
|  | *Rosa chinensis* | AVR48789.1 | germacrene D synthase |  |
|  | *Citrus sinensis* | AAQ04608.1 | valencene synthase |  |
|  | *Cichorium intybus* | AAM21658.1 | germacrene A synthase |  |
|  | *Lavandula angustifolia* | AGL98420.1 | germacrene-D synthase |  |
|  | *Lavandula angustifolia* | AGL98419.1 | caryophyllene synthase |  |
|  | *Lavandula angustifolia* | ABB73046.1 | bergamotene synthase |  |
|  | *Lavandula viridis* | AGN72806.1 | germacrene A |  |
|  | *Lavandula stoechas* | AGN72803.1 | germacrene A |  |
|  | *Lavandula pedunculata* | AGN72800.1 | germacrene A |  |
| TPS-b | *Citrus limon* | AAM53943.1 | gamma-terpinene synthase |  |
|  | *Artemisia annua* | AAK58723.1 | (-)-beta-pinene synthase |  |
|  | *Salvia officinalis* | AAC26018.1 | (+)-sabinene synthase |  |
|  | *Mentha longifolia* | AAD50304.1 | limonene synthase |  |
|  | *Arabidopsis thaliana* | AAG09310.1 | myrcene/ocimene synthase |  |
|  | *Arabidopsis thaliana* | AAN65379.1 | E-beta-ocimene synthase |  |
|  | *Populus tremula* x*Populus alba* | CAC35696.1 | isoprene synthase |  |
|  |  |  |  |  |
| TPS fimaly | Species | Protein ID/Accession | Note |  |
|  | *Lavandula angustifolia* | ABB73045.1 | linalool synthase |  |
|  | *Lavandula angustifolia* | ABB73044.1 | limonene synthase |  |
|  | *Lavandula angustifolia* | AFL03423.1 | 1,8-cineole synthase |  |
|  | *Lavandula viridis* | AGN72804.1 | alpha fenchol |  |
|  | *Lavandula pedunculata* | AGN72799.1 | alpha pinene |  |
|  | *Lavandula stoechas* | AGN72802.1 | alpha pinene |  |
|  | *Lavandula* x *intermedia* | AXF50410.1 | linalool synthase |  |
|  | *Lavandula* x *intermedia* | ARA91314.1 | carene synthase |  |
|  | *Lavandula* *angustifolia* | ADQ73631.1 | phellandrene synthase |  |
|  | *Lavandula* x *intermedia* | AGU13712.1 | caryophyllene synthase |  |
|  | *Lavandula* x *intermedia* | AFL03421.1 | 1,8-cineole synthase |  |
|  | *Lavandula viridis* | AGN72805.1 | alpha pinene |  |
|  | *Lavandula latifolia* | ABD77417.1 | linalool synthase |  |
|  | *Lavandula latifolia* | AFL03422.1 | 1,8-cineole synthase |  |
|  | *Lavandula pedunculata* | AGN72799.1 | alpha pinene |  |
| TPS-c | *Cistus creticus subsp. creticus* | ADJ93862.1 | copal-8-ol diphosphate synthase |  |
|  | *Helianthus annuus* | CBL42915.1 | copalyldiphosphate synthase |  |
|  | *Oryza sativa* | Q6ET36.1 | Ent-copalyl diphosphate synthase 1 |  |
|  | *Oryza sativa* | Q6Z5I0.1 | Ent-copalyl diphosphate synthase 2 |  |
|  | *Oryza sativa* | Q6E7D7.1 | Syn-copalyl diphosphate synthase |  |
|  | *Arabidopsis thaliana* | AAA53632.1 | ent-kaurene synthetase A |  |
|  | *Cucurbita maxima* | AAD04292.1 | copalyl diphosphate synthase 1 |  |
|  | *Pisum sativum* | AAB58822.1 | ent-kaurene synthase A |  |
|  | *Solanum lycopersicum* | BAA84918.1 | copalyl diphosphate synthase |  |
| TPS-d | *Picea abies* | AAS47694.1 | (-)-limonene synthase |  |
|  | *Picea abies* | AAS47692.1 | (-)-alpha/beta-pinene synthase |  |
|  | *Picea abies* | AAS47691.1 | levopimaradiene/abietadiene synthase |  |
|  |  |  |  |  |
|  |  |  |  |  |
| TPS fimaly | Species | Protein ID/Accession | Note |  |
|  | *Picea sitchensis* | AAP72020.1 | pinene synthase |  |
|  | *Pinus taeda* | AAO61228.1 | (+)-alpha-pinene synthase |  |
|  | *Pinus taeda* | AAO61227.1 | alpha-terpeniol synthase |  |
|  | *Abies grandis* | AAF61453.1 | beta-phellandrene synthase |  |
|  | *Abies grandis* | AAC05727.1 | δ-selinene synthase |  |
|  | *Ginkgo biloba* | AAL09965.1 | levopimaradiene synthase |  |
|  | *Taxus brevifolia* | AAC49310.1 | taxadiene synthase |  |
| TPS-e/f | *Clarkia breweri* | AAC49395.1 | S-linalool synthase |  |
|  | *Arabidopsis thaliana* | Q93YV0.1 | terpenoid synthase 4 |  |
|  | *Cucurbita maxima* | AAB39482.1 | ent-kaurene synthase B |  |
|  | *Arabidopsis thaliana* | AAC39443.1 | ent-kaurene synthase |  |
|  | *Helianthus annuus* | CBL42917.1 | kaurene synthase |  |
|  | *Scoparia dulcis* | AEF33360.2 | ent-kaurene synthase |  |
|  | *Oryza sativa*Japonica Group | AAQ72559.1 | ent-kaurene synthase 1A |  |
|  | *Oryza sativa* Indica Group | AAU05906.1 | syn-pimara-7,15-diene synthase |  |
|  | *Grindelia hirsutula* | AGN70888.1 | geranyl linalool synthase |  |
|  | *Oryza sativa* Japonica Group | BAD34478.1 | stemer-13-ene synthase |  |
| TPS-g | *Ocimum basilicum* | AAV63789.1 | R-linalool synthase |  |
|  | *Antirrhinum majus* | AAO41726.1 | myrcene synthase Oc15 |  |
|  | *Arabidopsis thaliana* | NP_176361.2 | terpene synthase 14 |  |
|  | *Antirrhinum majus* | ABR24417.1 | nerolidol/ linalool synthase 1 |  |
|  | *Antirrhinum majus* | AAO41727.1 | myrcene synthase 1e20 |  |
|  | *Vitis vinifera* | ADR74218.1 | geraniol synthase |  |
|  | *Catharanthus roseus* | AFD64744.1 | geraniol synthase |  |
|  | *Ocimum basilicum* | AAR11765.1 | geraniol synthase |  |
|  | *Olea europaea* | AFI47926.1 | geraniol synthase 1 |  |
|  |  |  |  |  |
|  | |  |  |  |
| TPS fimaly | Species | Protein ID/Accession | Note |  |
| TPS-h | *Selaginella moellendorffii* | XP_002960350.2 | copalyl diphosphate synthase 1 |  |
|  | *Selaginella moellendorffii* | XP_024527840.1 | copalyl diphosphate synthase 2 |  |
|  | *Selaginella moellendorffii* | XP_002967364.2 | copalyl diphosphate synthase 2-like |  |
