# Supplementary material for: Terpene produced by coexpression of the TPS and P450 genes from Lavandula angustifolia protects plants from herbivore attacks during budding stages
Source: BMC Plant Biol. 2023 Oct 9;23:477. doi: 10.1186/s12870-023-04490-7 (PMC10561503; doi:10.1186/s12870-023-04490-7)
Supplement: Supplementary file 4 — Supplementary Material 4 [file 12870_2023_4490_MOESM4_ESM.docx]

**Table S3. Amino acid sequences used for building CYP phylogenetic tree**

| family | Species | | Protein ID/Accession | Protein ID/Accession | Note |
| --- | --- | --- | --- | --- | --- |
| CYP51G1 | *Arabidopsis thaliana* | | NP_172633.1 | NP_172633.1 | CYP51G1 |
| CYP51 | *Hevea brasiliensis* | | AIU41378.1 | AIU41378.1 | CYP51 |
| CYP51 | *Sorghum bicolor* | | AAC49659.1 | AAC49659.1 | 14-alpha demethylase |
| CYP51 | *Nicotiana tabacum* | | AAL54888.1 | AAL54888.1 | obtusifoliol-14-demethylase |
| CYP51 | *Solanum chacoense* | | AAT12274.1 | AAT12274.1 | obtusifoliol 14alpha-demethylase |
| CYP51 | *Petunia* x *hybrida* | | CAL69911.1 | CAL69911.1 | obtusifoliol-14-demethylase |
| CYP51 | *Solanum lycopersicum* | | ADJ37071.1 | ADJ37071.1 | obtusifoliol 14alpha-demethylase |
| CYP51 | *Solanum lycopersicum* | | NP_001234537.2 | NP_001234537.2 | sterol C14-demetylase |
| CYP51 | *Fritillaria cirrhosa* | | APU50920.1 | APU50920.1 | obtusifoliol 14-alpha demethylase |
| CYP51 | *Pisum sativum* | | BAR45707.1 | BAR45707.1 | cytochrome monooxygenase like |
| CYP71A1 | *Fritillaria cirrhosa* | | APU50916.1 | APU50916.1 | FcCYP71A1-1 |
| CYP71A1 | *Persea americana* | | AAA32913.1 | AAA32913.1 | PaCYP71A1 |
| CYP71A1 | *Pyrus communis* | | AAL66194.1 | AAL66194.1 | PcCYP71A1 |
| CYP71AV8 | *Cichorium intybus* | | ADM86719.1 | ADM86719.1 | valencene oxidase |
| CYP71A32 | *Mentha* x *piperita* | | AAL06397.1 | AAL06397.1 | menthofuran synthase |
| CYP71BA1 | *Zingiber zerumbet* | | BAJ39893.1 | BAJ39893.1 | ZzCYP71BA1 |
| CYP71BL1 | *Helianthus annuus* | | F8S1H3.1 | F8S1H3.1 | Germacrene A acid 8-beta-hydroxylase |
| CYP71BL2 | *Lactuca sativa* | | F8S1I0.1 | F8S1I0.1 | Costunolide synthase |
| CYP71BE5 | *Vitis vinifera* | | XP_010644548.1 | XP_010644548.1 | premnaspirodiene oxygenase-like |
| CYP71D12 | *Catharanthus roseus* | | P98183.2 | P98183.2 | Tabersonine 16-hydroxylase |
| CYP71D13/15 | *Mentha* x *piperita* | | Q9XHE7.1 | Q9XHE7.1 | (-)-(4S)-limonene-3-hydroxylase |
| CYP71D16 | *Nicotiana tabacum* | | AAD47832.1 | AAD47832.1 | CBT-ol to CBT-diol |
| CYP71D18 | *Mentha spicata* | | Q9XHE8.1 | Q9XHE8.1 | (4S)-limonene-6-hydroxylase |
| CYP71D20 | *Nicotiana tabacum* | | NP_001311564.1 | NP_001311564.1 | 5-epiaristolochene-1,3-dihydroxylase |
| CYP71D55 | *Hyoscyamus muticus* | | A6YIH8.1 | A6YIH8.1 | Premnaspirodiene oxygenase |
|  |  | |  |  |  |
|  | |  |  |  |  |
| family | Species | | Protein ID/Accession | Protein ID/Accession | Note |
| CYP72A1 | *Perilla frutescens* | | Q05047.1 | Q05047.1 | Secologanin synthase |
| CYP72A67 | *Medicago truncatula* | | ABC59075.1 | ABC59075.1 | MtCYP72A67 |
| CYP72A154 | *Mucuna pruriens* | | RDX90634.1 | RDX90634.1 | [11-oxo-beta-amyrin-30-oxidase](https://www.ncbi.nlm.nih.gov/protein/RDX90634.1) |
| CYP76B6 | *Catharanthus roseus* | | Q8VWZ7.1 | Q8VWZ7.1 | Geraniol-10-hydroxylase |
| CYP76B10 | *Swertia mussotii* | | D1MI46.1 | D1MI46.1 | Geraniol-8-hydroxylase |
| CYP88A1 | *Zea mays* | | NP_001105586.1 | NP_001105586.1 | ent-kaurenoic acid oxidase |
| CYP88D6 | *Glycyrrhiza uralensis* | | B5BSX1.1 | B5BSX1.1 | Beta-amyrin-11-oxidase |
| CYP93E1 | *Glycine max* | | NP_001236154.2 | NP_001236154.2 | Beta-amyrin-24-hydroxylase |
| CYP93E2 | *Medicago truncatula* | | ABC59085.1 | ABC59085.1 | Balsams alcohol-11-hydroxylase |
| CYP93E3 | *Glycyrrhiza uralensis* | | BAG68930.1 | BAG68930.1 | Balsams alcohol-11-hydroxylase |
| CYP97A3 | *Arabidopsis thaliana* | | Q93VK5.1 | Q93VK5.1 | Carotenoids *β*-hydroxylase |
| CYP97C1 | *Arabidopsis thaliana* | | Q6TBX7.1 | Q6TBX7.1 | Carotenoid ε-ring hydroxylase |
| CYP97C2 | *Oryza sativa* | | XP_015613023.1 | XP_015613023.1 | carotene epsilon-monooxygenase |
| CYP97H1 | *Euglena gracilis* | | BAX73989.1 | BAX73989.1 | carotene beta-ring hydroxylase |
| CYP701A1/  A10/A13 | *Cucurbita maxima* | | AAG41776.1 | AAG41776.1 | ent-kaurene oxidase |
| CYP701A3 | *Arabidopsis thaliana* | | NP_197962.1 | NP_197962.1 | GA requiring 3 |
| CYP707A3 | *Arabidopsis thaliana* | | Q9FH76.1 | Q9FH76.1 | Abscisic acid 8'-hydroxylase 3 |
| CYP714D1 | *Oryza sativa* | | Q5KQH7.1 | Q5KQH7.1 | Gibberellic acid catabolism |
| CYP714A2 | *Arabidopsis thaliana* | | Q6NKZ8.1 | Q6NKZ8.1 | EUI-like P450 A2 |
| CYP716A12 | *Medicago truncatula* | | Q2MJ20.1 | Q2MJ20.1 | Beta-amyrin-28-monooxygenase |
| CYP716A179 | *Glycyrrhiza uralensis* | | BAW34647.1 | BAW34647.1 | triterpene C-28 oxidase |
| CYP716A1 | *Arabidopsis lyrata* | | EFH44718.1 | EFH44718.1 | AlCYP716A1 |
| CYP720B1 | *Pinus taeda* | | Q50EK6.1 | Q50EK6.1 | Abietadienol/abietadienal oxidase |
| CYP720B2 | *Pinus taeda* | | Q50EK5.1 | Q50EK5.1 | PtCYP720B2 |
|  |  | |  |  |  |
|  | |  |  |  |  |
| family | Species | | Protein ID/Accession | Protein ID/Accession | Note |
| CYP720B12 | *Pinus contorta* | | AIK01734.1 | AIK01734.1 | PcCYP720B12 |
| CYP720B4 | *Picea sitchensis* | | ADR78276.1 | ADR78276.1 | PsCYP720B4 |
| CYP725A1 | *Taxus cuspidata* | | Q9AXM6.1 | Q9AXM6.1 | Taxane 10-beta-hydroxylase |
| CYP725A2 | *Taxus cuspidata* | | Q8W4T9.1 | Q8W4T9.1 | Taxane 13-*α*-hydroxylase |
| CYP725A3 | *Taxus cuspidata* | | Q84KI1.1 | Q84KI1.1 | Taxane 14-*β*-hydroxylase |
| CYP735A1/A2/  A3/A4 | *Arabidopsis thaliana* | | Q9FF18.1\| | Q9FF18.1\| | Cytokinin hydroxylase |
| GAO1 | *Lactuca sativa* | | D5J9U8.1 | D5J9U8.1 | Germacrene A hydroxylase |
| GAO2 | *Cichorium intybus* | | D5JBW8.1 | D5JBW8.1 | Germacrene A hydroxylase |
| GAO3 | *Saussurea costus* | | D5JBW9.1 | D5JBW9.1 | Germacrene A hydroxylase |
| GAO4 | *Helianthus annuus* | | XP_022000663.1 | XP_022000663.1 | Germacrene A oxidase |
| GAO5 | *Barnadesia spinosa* | | D5JBX1.1 | D5JBX1.1 | Germacrene A hydroxylase |
| CYP76C3 | *Arabidopsis thaliana* | | AEC10573.1 | AEC10573.1 | AtCYP76C3 |
| CYP71B31 | *Arabidopsis thaliana* | | OAP05543.1 | OAP05543.1 | AtCYP71B31 |
| CYP76C2 | *Arabidopsis thaliana* | | OAP07869.1 | OAP07869.1 | AtCYP76C2 |
| CYP76C4 | *Arabidopsis thaliana* | | OAP09091.1 | OAP09091.1 | AtCYP76C4 |
| CYP76C1 | *Arabidopsis thaliana* | | OAP07350.1 | OAP07350.1 | AtCYP76C1 |
| CYP726A14 | *Ricinus communis* | | NP_001310623.1 | NP_001310623.1 | Premnaspirodiene oxygenase 1 |
| CYP726A17 | *Ricinus communis* | | NP_001310626.1 | NP_001310626.1 | Premnaspirodiene oxygenase 4 |
| CYP726A18 | *Ricinus communis* | | NP_001310627.1 | NP_001310627.1 | Premnaspirodiene oxygenase 5 |
| CYP726A16 | *Ricinus communis* | | NP_001310625.1 | NP_001310625.1 | Premnaspirodiene oxygenase 3 |
| CYP726A15 | *Ricinus communis* | | NP_001310624.1 | NP_001310624.1 | Premnaspirodiene oxygenase 2 |
| CYP76AK6 | *Salvia fruticosa* | | AOW42544.1 | AOW42544.1 | 11-hydroxyferruginol C20-oxidase |
| CYP76AK7 | *Salvia rosmarinus* | | AOW42545.1 | AOW42545.1 | 11-hydroxyferruginol C20-oxidase 1 |
| CYP76AK8 | *Salvia rosmarinus* | | AOW42546.1 | AOW42546.1 | 11-hydroxyferruginol C20-oxidase 2 |
| CYP76AH1 | *Salvia miltiorrhiza* | | S4UX02.1 | S4UX02.1 | Ferruginol synthase |
| family | Species | | Protein ID/Accession | Protein ID/Accession | Note |
| CYP93E2 | *Medicago truncatula* | | ABC59085.1 | ABC59085.1 | cytochrome P450 monooxygenase |
| CYP76AH24 | *Salvia pomifera* | | ALM25796.1 | ALM25796.1 | SpCYP76AH24-like |
| CYP87D16 | *Maesa lanceolata* | | AHF22090.1 | AHF22090.1 | MLCYP87D16 |
| CYP71D374 | *Salvia miltiorrhiza* | | AJD25162.1 | AJD25162.1 | SmCYP71D374 |
| CYP71D378 | *Plectranthus barbatus* | | AMZ03380.1 | AMZ03380.1 | PbCYP71D378 |
| CYP71D377 | *Plectranthus barbatus* | | AMZ03383.1 | AMZ03383.1 | PbCYP71D377 |
| CYP71D95 | *Mentha spicata* | | Q6IV13.1 | Q6IV13.1 | limonene-3-hydroxylase |
